# Paxillin is crucial for thymus and parathyroid development by regulating the architecture of the third pharyngeal pouch endoderm

**DOI:** 10.1007/s00018-025-05973-6

**Published:** 2026-01-26

**Authors:** O. Iacolare, M. Bilio, A. Altomonte, O. Lanzetta, C. Turner, A. Baldini, D. Alfano

**Affiliations:** 1 CNR-Institute of Genetics and Biophysics Adriano Buzzati-Traverso, Via Pietro Castellino, Naples, Italy; 2 Department of Cell and Developmental Biology, State University of New York Upstate Medical University, Syracuse, NY USA; 3 Department of Molecular Medicine and Medical Biotechnology, University of Naples Federico II, Naples, Italy; 4 Present Address: Departments of Experimental Medicine and Internal Medicine, University of Genoa, Genoa, Italy

**Keywords:** Pharyngeal development, Pharyngeal pouch, Paxillin, Tbx1, DiGeorge syndrome

## Abstract

The paxillin (PXN) protein is a key component of focal adhesions in which it primarily functions as a molecular scaffold to spatiotemporally integrate diverse signalling networks to transduce intracellular responses. In this study, using loss-of-function genetics in mice, we investigated whether the *Pxn* gene has a role in the morphogenesis of the pharyngeal apparatus and whether it interacts with the *Tbx1* gene, which encodes a key transcription factor required in pharyngeal development. Conditional deletion of *Pxn* in the *Tbx1* expression domain did not cause cardiac defects. Instead, the germline *Pxn* mutation led to cardiac anomalies and to morphogenetic defects of the third pharyngeal pouch (3PP). We found that in *Pxn* deleted embryos, the 3PP was hypoplastic, lacked the expression of *Gcm2*, a gene that marks the parathyroid domain, but expressed *FoxN1*, a gene marking the thymic domain. Consistently, the parathyroids did not develop, and the thymus was hypoplastic and/or malpositioned. The reduced dosage of *Tbx1* had a more severe effect on cardio-pharyngeal defects of *Pxn*^−/−^ embryos, suggesting an interaction between *Pxn* and *Tbx1*. Thus, a novel function of Pxn in the control of parathyroid and thymic development has been discovered, probably through a function in the pouch endoderm, and intriguingly we found a strong genetic interaction between *Tbx1* and *Pxn*.

**Graphical Abstract:**

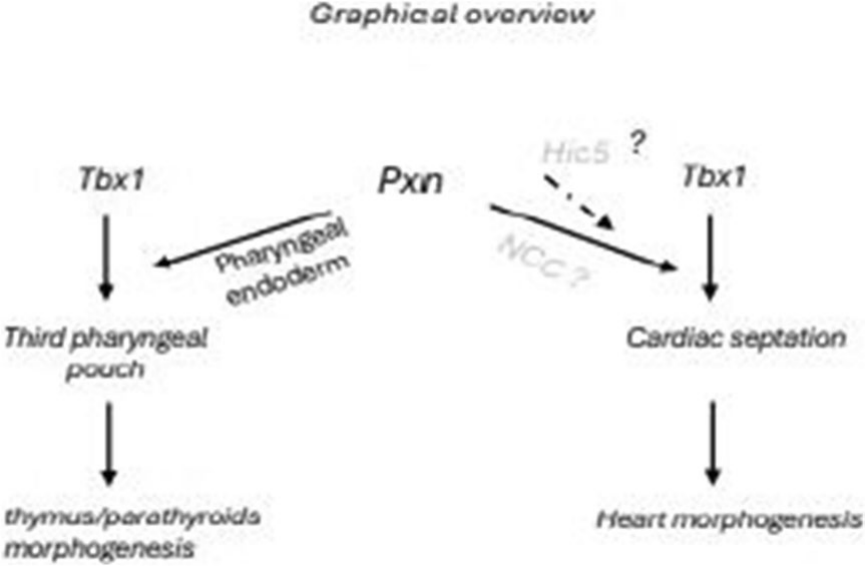

**Supplementary Information:**

The online version contains supplementary material available at 10.1007/s00018-025-05973-6.

## Introduction

Paxillin (PXN) is a key component of focal adhesions (FAs) in which it primarily functions as a molecular scaffold to spatiotemporally integrate diverse signalling networks, transducing and coordinating dynamic intracellular responses to a variety of stimuli. Through its interactome, PXN has been shown to regulate FA growth, stabilization, and disassembly, enabling migration on 2D surfaces as well as invasion through 3D-ECM [1, 2]. While the FA adapter protein Pxn is a well-characterized regulator of cell adhesion signaling, F-actin cytoskeleton remodelling and single cell migration, its role in in vivo epithelial tissue organization is poorly known [3] and its contribution in in vivo heart morphogenesis has not been deeply investigated; in fact, only Hagel et al. showed that *Pxn* is indispensable and its loss causes embryonic lethality at E9.5 and that *Pxn* knockout mice had defects in multiple mesodermally derived structures such as heart and somites [4]. It is known that Pxn (as well as the focal adhesion pathway) is essential for mesodermal cell motility in a non-canonical Wnt pathway manner in late phase of gastrulation [5, 6]. We previously found that *Pxn* is a target of the TBX1 transcription factor; in fact, analyses of genome-wide target gene data suggested that loss or reduced dosage of *Tbx1* unexpectedly perturbs gene pathways related to FA dynamics, extracellular matrix-receptor interaction, cell movement, and other determinants of cell morphology [7–9]. Very recently we found that TBX1 regulates FA by mainly influencing their disassembly process and by modulating Paxillin-mediated signalling and integrin trafficking [10]. We have identified a novel function of Tbx1 in regulating the extracellular-cell interaction within the SHF, particularly by downregulating *Pxn* [11]. We found that loss of Tbx1 impairs the ECM-integrin-FA pathway; interfering with the ECM-integrin-FA axis in a mouse embryos culture model by using a specific inhibitor caused OFT defects [11]. In particular, the specific inhibitor 6-B345TTQ, which blocks the alpha4 integrin-PXN interaction, caused OFT shortening. *Tbx1* is an important player in the development of the SHF, cardio-pharyngeal mesoderm and the pharyngeal endoderm. The gene is strongly implicated in DiGeorge/22q11.2 deletion syndrome, a developmental disorder that affects the cardio-pharyngeal apparatus [12].

In this study, we tested whether *Pxn* has a role in the development of the pharyngeal apparatus (PA) and also whether *Pxn* and *Tbx1* interact during this process. We discovered that *Pxn* loss caused cardiac and pharyngeal defects and interestingly, we also found a *Pxn*—*Tbx1* interaction affecting the third pharyngeal pouch endoderm development and resulting in thymic and parathyroid hypoplasia or aplasia.

## Results

### Deletion of Pxn in the mesoderm alters epithelial marker expression, but does not cause cardiac defects

**Fig. 1:**
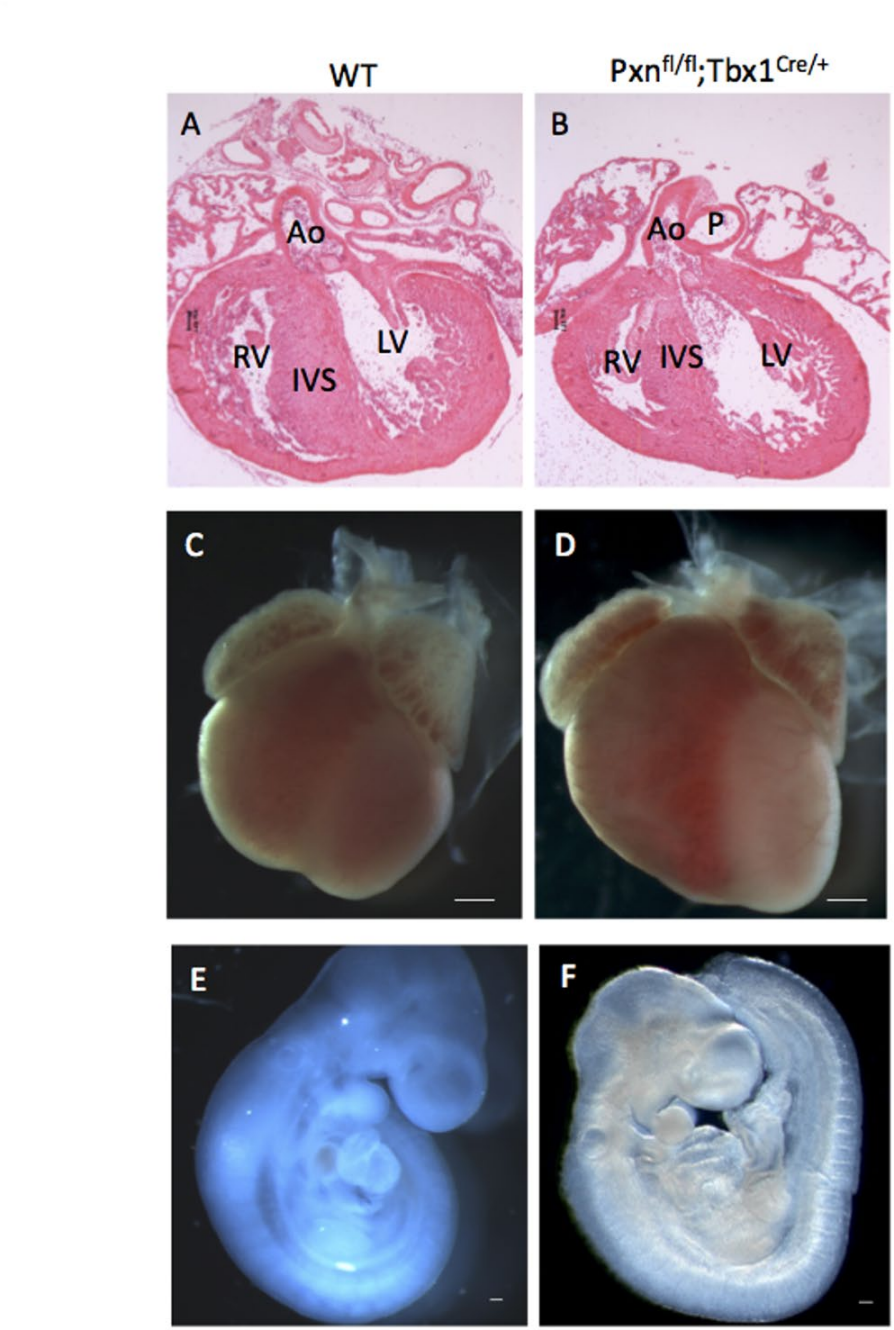
Pxn loss in mesodermal territories does not cause heart anomalies. (**A-B**) Eosin-stained sections or (**C-D**) whole hearts revealing that E18.5 conditional *Pxn*^*fl/fl*^*;Tbx1-Cre* mutants appear normal as the control hearts (*WT*). (**E–F**) Right views of embryonic day E 9.5 *Pxn*^*fl/fl*^*;Tbx1*^*Cre/*+^ *and WT* whole embryos displaying normal cardiac development. IVS, interventricular septum; RV, right ventricle; LS, left ventricle. Scale bars, 100 µm

*Pxn* is expressed in the embryonic mesoderm [4], moreover, it is expressed in the second heart field (SHF), a population of cardiac progenitors that contributes to the outflow tract of the heart [11]. *Pxn* is a target of TBX1, one of the most crucial transcriptional factors of cardiac development, having a critical role in the cardiopharyngeal lineage, as well as in maintaining cardiopharyngeal mesoderm (CPM) transcriptional identity and driving morphogenesis in the pharyngeal apparatus [9]. *Tbx1* is an important regulator of the earliest events in heart development and thymus organogenesis, the latter occurs prior to overt organ development and relates to molecular control of third and fourth pharyngeal pouch formation [13]. These findings led us to ask whether *Pxn* is crucial for heart morphogenesis; therefore, we deleted *Pxn* by using *Pxn*^*flox*/flox^ animals [3, 14] and the *Tbx1*^*Cre*^ driver [15]. We crossed *Pxn*^*flox/*+^*;Tbx1*^*Cre/*+^ and *Pxn*^*flox/*+^ animals and examined 10 *Tbx1*^*Cre/*+^*;Pxn*^*flox/flox*^ embryos at E 9.5 and E18.5 but could not find any cardiac anomaly (Fig. 1). Moreover, we conditionally deleted Pxn with two more Cre drivers: *Mef2c-AHF-Cre* [16] and *Mesp1*^*Cre*^ [17] expressed in the SHF and in the entire anterior mesoderm, respectively. Consistently, E9.5 and E18.5, *Pxn*^*flox/flox*^*;Mef2c-*^*AHF−Cre/*+^ and *Pxn*^*flox/flox*^*;Mesp1*^*Cre/*+^ embryos did not show any cardiac morphogenetic defect (Supplementary Fig. 1A). At the cellular level, in *Pxn*^*flox/flox*^*;Mef2c-*^*AHF−Cre/*+^ we observed an expansion of the E-cadherin domain, accumulating from the anterior to the posterior SHF (Supplementary Fig. 1B-C), in contrast to control embryos in which it was restricted to the anterior SHF; also, in these mutants, the integrin beta-1 signal was restricted to the lateral side of eSHF cells (SHF cells that hold epithelial properties, [18]) rather than being distributed through all their circumferential membrane (including both apico-basal and lateral domains). These results are consistent with the established in vitro role of Pxn in regulating cell–cell adhesion [19]. On the other hand, in an in vitro epithelial culture system, we observed that *Tbx1* loss caused an alteration of E-cadherin distribution compared to control cells (Supplementary Fig. 2), suggesting that TBX1, possibly through PXN, is able to regulate cell–cell adhesion structures. Recently, it has been shown that paxillin is required for AJ assembly through facilitating E-cadherin endocytosis [20].

**Fig. 2:**
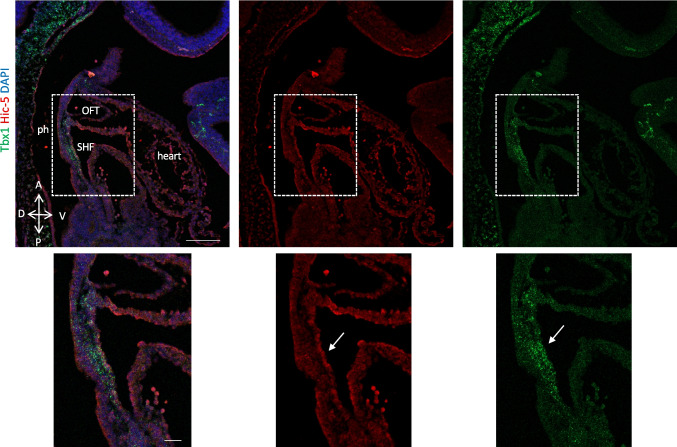
Hic5 is expressed in the SHF. RNAscope in situ hybridization with Tbx1 mRNA probe (red) and anti-Hic5 antibody (red) on sagittal sections from wild-type embryos at E9.5. The white dotted line indicates the position in images with higher magnification. Scale bar, 100 µm. ph, pharynx; SHF, second heart field; OFT, outflow tract; nt, neural tube

Functional redundancy between *Pxn* and the Pxn-family member *Tgfb1i1* (a.k.a. *Hic5*, hereafter referred to with this name) has been reported in regulating the integrin biology in different systems [21, 22]. In order to explain the lack of cardiac defects following conditional *Pxn* ablation in the anterior mesoderm, we we asked whether *Hic5* is expressed in the SHF by performing two-color RNAscope in situ hybridization analysis using a probe for *Tbx1*; interestingly, we found that at E9.5, *Hic5* is expressed in the eSHF (Fig. 2; Supplementary Fig. 3). Thus, it is possible that the absence of cardiac defects might be due to functional redundancy in the SHF. We also tested the expression of the two proteins by immunofluorescence in E9.5 embryos and we found that PXN and HIC5 partially overlap in the pharyngeal pouch endoderm, and in the SHF and OFT (Supplementary Fig. 6), supporting the hypothesis of functional redundancy.

We next investigated whether the expression patterns of Hic5 and Tbx1 overlap. Hic5 expression in E9.5 mouse embryos was low in the first, second, and third pharyngeal arches (PAs) and their corresponding pharyngeal pouches (PPs), but higher in epithelial-like cells of the anterior second heart field (SHF)/dorsal pericardial wall (DPW), where it overlaps with Tbx1. In the PAs, HIC5 did not colocalize with TBX1; instead, it was mostly expressed in endothelial cells and few surrounding mesenchymal cells, while Tbx1 was predominantly localized to the core mesoderm (Supplementary Fig. 7–9). The expression of Hic5 in the SHF was also confirmed by published single-cell RNA sequencing data [23], Fig. 5C).

### Germline loss of *Pxn* leads to thymus, parathyroid and heart defects

**Fig. 3:**
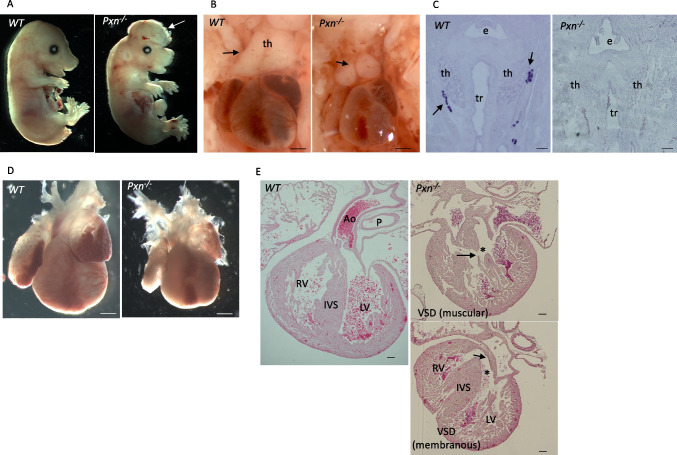
Pxn^*KO*^ mutant shows defects of pharyngeal endoderm-derived glands and cardiac anomalies. **A** Right views of E18.5 Pxn^*KO*^ (Pxn^−/−^) embryos showing that are smaller compared to control (WT), they show exencephaly (arrow). **B** the thymus appears hypoplastic or ectopic, with lobes rounded and not pyramidal in shape. **C** Coronal sections of E16.5 embryos by in situ hybridization with *Gcm2* showed the absence of parathyroid gland in *Pxn*^*−/−*^ embryos. n. = 3. Scale bars, 200 µm. E, esophagus; tr, trachea; ty, thyroid gland. **D** E 18.5 hearts appeared smaller compared to control. **E** Eosin-stained sections of E16.5 hearts revealing that Pxn^*KO*^ mutants (Pxn^−/−^) display congenital heart defects including an overriding aorta (asterisk) and membranous and/or muscular ventricular septal defect (arrows). Scale bars, 100 µm. th, thymus; tr, thyroid; P, pulmonary trunk; Ao, aorta; RV, right ventricle; LV, left ventricle; IVS, intraventricular septum and VSD, ventricular septal defect

These findings led us to investigate the effects of germline Pxn deletion, given that the role of *Pxn* in the development of the pharyngeal apparatus has not been investigated. In fact, the only data in the literature are derived from studies of Hagel and colleagues showing that *Pxn*^*−/−*^ mice had defects in multiple mesodermally derived structures such as heart and that they die at E9.5 [4]*.* To generate mice carrying germline Pxn deletion, we crossed *Pxn*^*flox/flox*^ mice with *Mef2c-AHF-Cre* females (a universal deleter strain when transmitted through the female germ line) to generate *Pxn*^±^ mice; the line was crossed with WT animals for at least two generations to eliminate possible mosaicism. *Pxn*^−/−^ embryos (E15.5-E18.5), obtained by inter-crossing *Pxn*^±^ mice, were recovered at the expected Mendelian ratio (20/80, 25%) and did not show any PXN expression (Suppl. Figure 4A-B). *Pxn*^−/−^ embryos were smaller, and showed severe exencephaly (100% penetrance *n* = 30) and the more severe cases had anophthalmia (Fig. 3A and Supplementary Fig. 4C); the thymus appeared hypoplastic compared to WT controls (*n* = 5/12, 41% penetrance); lobes were rounded rather pyramidal in shape, and in a small percentage it had a single lobe (Fig. 3B). We could not detect any parathyroid tissue in the entire mediastinic region in coronal and transverse sections of 5 E16.5 embryos by in situ hybridization with *Gcm2* (Fig. 3C). Additionally, we found cardiac ventricular septal defects (VSDs) of muscular and/or perimembranous type in 6 of the 11 embryos examined (54.5%) (Fig. 3D and E), and also atrial septal defects (ASD) (37.5%); we could not find other morphological defects in mutant hearts.

**Fig. 4:**
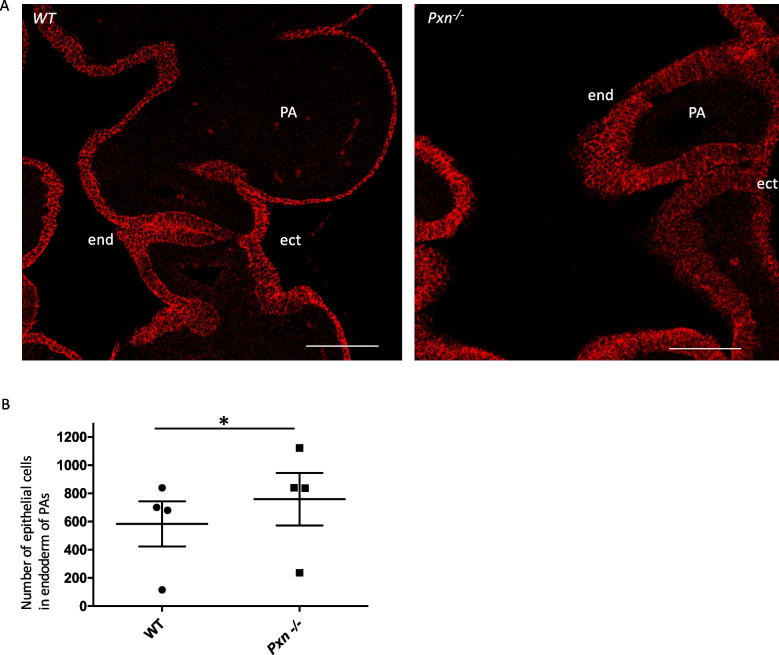
Endoderm epithelial accumulation within the PA of Pxn mutant. **A** E10.5 coronal sections showing E-cadherin positive epithelial cells delimiting pharyngeal arches. In the Pxn^−/−^ mutant the endodermal epithelial cells are multilayered compared to control *WT* embryos. Scale bar, 50 µm. **B** Quantification of endoderm stratification within the pharyngeal arches by counting the number of E-cadherin positive cells. *n* = 4. PA, pharyngeal arch; end, endoderm; ect, ectoderm. *p* = 0.017*.* ec, ectoderm; end, endoderm

We found that in *Pxn*^*−/−*^ embryos, the pharyngeal epithelial organization was altered; in fact, excessive multilayered epithelium occurred in the PA of Pxn mutants, where we found multilayered endoderm stratification, especially at the points where the cells would be turning inwards to invaginate (Fig. 4; Supplementary Fig. 5). This accumulation is not due to alteration of mitotic index, as suggested by the ratio of pH3 positive cells within the E-cadherin positive epithelial cells (in PE) (Supplementary Fig. 5D).

**Fig. 5:**
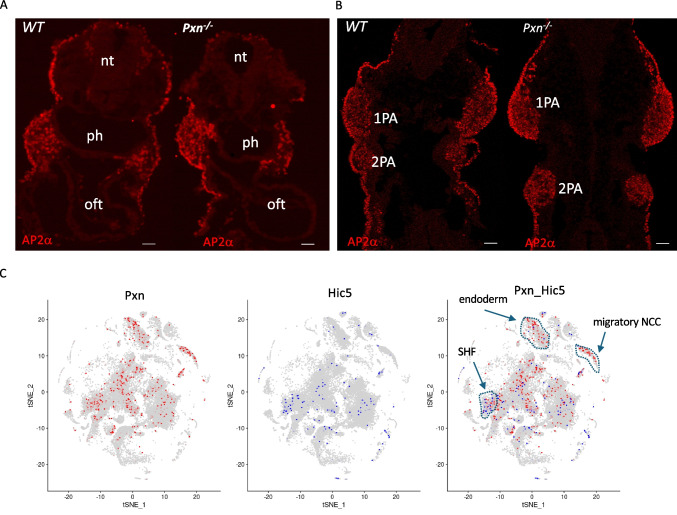
Paxillin espression pattern in E9.5 embryo. **A** Pxn and Ap2alpha immunostaining of transverse sections of E9.5 wt and Pxn^−/−^ embryos. Scale bar, 100 µm. **B** Feature plots representing the *Pxn* and *Hic5* single cells expression distributions obtained from atlas datasets. ph, pharynx; OFT, outflow tract; nt, neural tube; PA, pharyngeal arch

The PA is contributed by mesoderm, endoderm, ectoderm, and neural crest-derived cells (NCCs). In order to investigate the origin of the defects of the PA observed in *Pxn*^*−/−*^ mutants we also analysed the NCC population by IF with AP-2alpha antibodies, which mark migratory NCCs. However, the expression of this marker at E9.5 did not change between *Pxn*^*−/−*^ and control embryos both in the OFT and in the PAs (Fig. 5A and B). Exploratory data display from available atlas datasets [23] showed that *Pxn* and *Hic5* expression domains are mostly not-overlapping (Fig. 5C). In fact, *Pxn* seems to be highly expressed in pre-migratory NCC and endoderm territories, whereas *Hic5* in these regions is absent; in contrast, Hic5 is expressed in the SHF, where colocalize with PXN.

We also investigated the expression level of HIC5 in *Pxn*^*−/−*^ and *Tbx1*^*−/−*^ embryos and found that in 3 *Tbx1*^*−/−*^ embryos, HIC5 appears to be upregulated in the head mesenchyme, in the myocardial layer of the OFT, and in the myocardial layer of the atrium. In particular, atrioventricular endocardial cells stained with anti-Hic5 were more abundant in the mutant embryos compared to wild-type controls (Supplementary Fig. 10). In 3 *Pxn*^*−/−*^ embryos, Hic5 expression appeared increased in the vessel (both the pharyngeal arch arteries and the dorsal aorta) (Supplementary Fig. 10).

### *Tbx1* is a modifier of the *Pxn* mutants phenotype

**Fig. 6:**
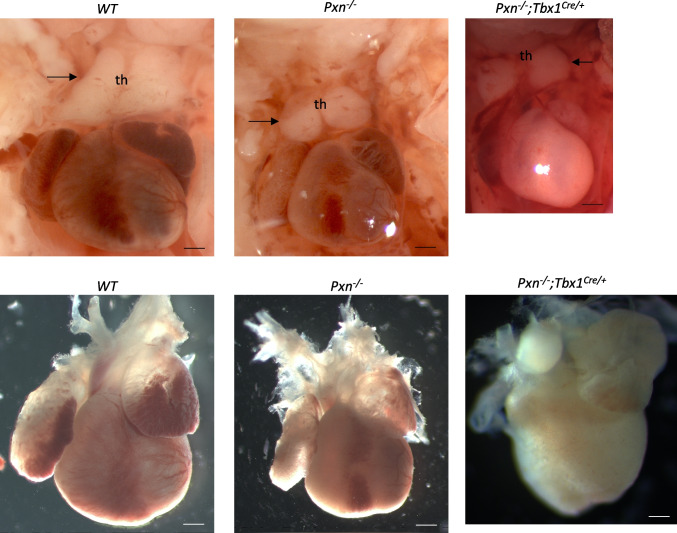
Tbx1 loss has a pejorative effect on heart and thymus defects of Pxn mutant embryos. Heart and thymus preparations from E18.5 embryos. Note the small thymus (th) in *Pxn*^*−/−*^*;Tbx1*^*Cre/*+^ compared to *Pxn*^*−/−*^ embryos. Scale bars, 150 µm

In order to understand whether *Tbx1* may be a modifier of *Pxn* mutant phenotypes, we crossed *Pxn*^±^ with *Tbx1*^*Cre/*+^ mice and we found that *Tbx1* heterozygosity causes a pejorative effect on cardiac and thymic defects because double heterozygous *Pxn*^±^*;Tbx1*^*Cre/*+^ exhibited a higher penetrance of VSD and higher expressivity of thymus hypoplasia compared to *Pxn*^±^ and *Tbx1*^*Cre/*+^ (Table 1; *p* = 0.017). In *Pxn*^*−/−*^*;Tbx1*^*Cre/*+^ embryos, thymus glands were more rostrally located than normal and were present at the same level of the embryos as the carotid arteries (Fig. 6). Hypoplastic thymus glands were smaller in size than normal glands (Fig. 6). At E15.5, double heterozygous *Pxn*^±^*;Tbx1*^*Cre/*+^ embryos had a higher incidence of thymic anomalies compared to Pxn^±^ or *Tbx1*^*Cre/*+^ embryos (Table 1).

**Table 1:** Phenotypes determined from E&E-stained coronal sections (heart defects) or whole mount images (thymic anomalies) of E16.5–18.5 embryos. *p* < 0.05. Pxn^±^;Tbx1^*Cre/*+^ double heterozygous vs Pxn^±^; Fisher's exact test

	Tb × 1 +/;P × n-/-	Tb × 1 ±;P × n-/-	Tb × 1 ±;P × n +/+	Tb × 1 +/; + P × n ±	Tb × 1 ±;P × n ±
N tot	12	14	17	23	32
N tot E&E	11	8	11	15	28
VSD (perimembranous or muscular)	6(54,5%)	6(75%)(*n.s*)	2(18%)	0%	9(32%)* p = 0.017
ASD	3/8(37,5%)	2/8(25%)	0/11(0%)	3/13(23%)	6/26(23%)
Thymus anomalies (hypoplastic or ectopic)	5(41%)	9(64%)	1(6%)	2(9%)	11(34%)

**Fig. 7:**
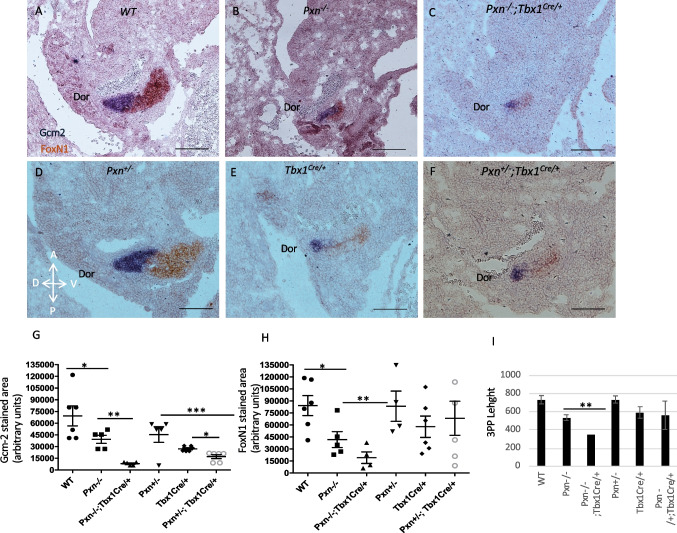
Tbx1 and Pxn genetically interact for 3PP morphogenesis. **A-F** Two-colour in situ hybridization of *Gcm2* (brown) and *FoxN1* (blue) in all genotypes analysed. **G** Graph showing quantitative analysis of *Gcm1*-positive area of 3PP of E11.5 embryos with different genotypes. **H** Graph showing quantitative analysis of *FoxN1*-positive area of 3PP of E11.5 embryos with different genotypes. **I** Graph representing quantitative analysis of 3PP size, measured as 3PP length. Dor, dorsal side of pouch. *p < 0.05; ** p < 0.01; *** p < 0.001.). Scale bar, 100 µm

To gain further insight into these findings, we analyzed phenotypic features of the 3PP, given that in the mouse, the thymus and parathyroid rudiments are derived from adjacent but distinct domains of the 3PP endoderm, and they express *FoxN1* (Forkhead box protein N1) and *Gcm2* (glial cells missing homolog 2) epithelial markers, respectively. *Tbx1* is expressed in the early endoderm that gives rise to both primordia but it is later turned off in the thymic domain [15, 24]. Therefore, we performed two-colour in situ hybridization of *Gcm2* (blue) and *FoxN1* (brown): in E11.5 control embryos, the staining spans the entire pouch, when the domains are clearly distinct and together the two markers stain the entire pouch (Fig. 7A). Pax1 was used as marker of PP (Supplementary Fig. 11). In *Pxn*^−/−^ embryos, the 3PP appeared to be smaller and rounder than in controls (Fig. 7B), where *FoxN1*-marked ventral domain and *Gcm2-*marked dorsal domain were barely detectable but correctly patterned. In *Pxn*^*−/−*^*;Tbx1*^*Cre/*+^ the expression of both markers was strongly reduced in intensity in 3PP in comparison to WT littermate controls (Fig. 7C). Moreover, the double heterozygous *Pxn*^±^*;Tbx1*^*Cre/*+^ embryos showed a statistically significant decrease in *Gcm2* expression in 3PP compared to *Pxn*^±^ or to *Tbx1*^*Cre/*+^ embryos, but no differences have been found in *FoxN1* expression (Fig. 7D, E and F).

When expression was quantified, both genes were significantly reduced in the 3PP region in *Pxn*^−/−^;*Tbx1*^*Cre/*+^ compared to *Pxn*^*−/−*^ embryos (** *p* values < 0.01.) (Fig. 7G and H). Instead, in the double heterozygous *Pxn*^±^*;Tbx1*^*Cre/*+^ embryos, only Gcm2 was significantly reduced in comparison to *Pxn*^±^ and *Tbx1*^*Cre/*+^ embryos (Fig. 7G and H). The presence of low expression of these two genes is consistent with the occurrence of mild thymus and parathyroid defects (hypolplastic thymus and/or ectopic parathyroids) in these embryos as compared to *Pxn* null mutant embryos. The length of 3PP appeared significantly smaller in double heterozygous *Pxn*^±^*;Tbx1*^*Cre/*+^ embryos in comparison to individual heterozygous embryos (Fig. 7I).

**Fig. 8:**
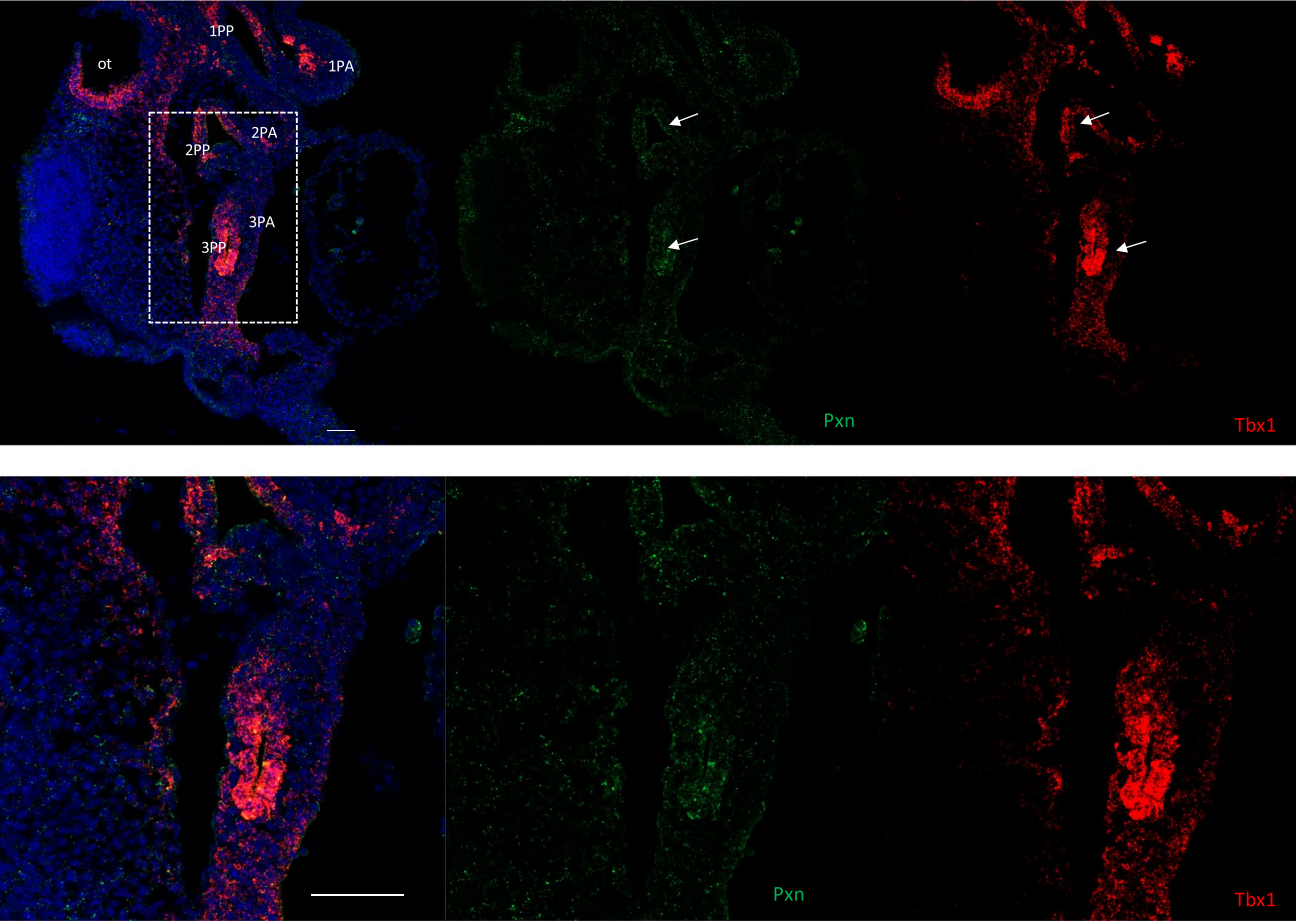
Tbx1 and Pxn overlap in the endoderm of the third pharyngeal pouch. **A** RNAscope in situ hybridization with Tbx1 mRNA probe (red) and Pxn mRNA (green) on sagittal sections from wild-type embryos at E9.5. Scale bar, 50 µm. **B** Images with high magnification corresponding to the dotted lines in panel A. Scale bar, 100 µm. ot, otocyst; PA, pharyngeal arch; PP, pharyngeal pouch

When we analyzed *Pxn* and *Tbx1* gene expression, we observed a partial overlap in the 3PP (and other PPs), in the PAs and in the SHF. Specifically, Pxn⁺ cells were scattered and more abundant in the proximal region of the archs, whereas Tbx1⁺ cells were mainly confined to the core mesoderm. The expression patterns of the two genes also showed areas of overlap in the head mesenchyme and pharyngeal endoderm (Fig. 8; Supplementary Fig. 12–17).

In addition, in reference single-cell RNASeq datasets [9, 25], both *Tbx1* and *Pxn* were detected in specific cell clusters, including undifferentiated mesodermal and epithelial cells (Supplementary Fig. 18).

Finally, we investigated whether the loss of *Pxn* affects *Tbx1* expression by quantifying its levels in the PAs and the SHF. Interestingly, a statistically significant increase of *Tbx1* expression was observed in *Pxn*⁻/⁻ embryos compared to controls (Supplementary Fig. 19).

## Discussion

In vivo, the role of *Pxn* has been explored only to a limited extent. Hagel et al. [4] reported that *Pxn*⁻/⁻ embryos died at embryonic day 9.5 (E9.5), displaying defects in multiple mesoderm-derived structures, including the heart. In contrast, our phenotypic analysis showed that conditional *Pxn* deletion in the mesoderm did not result in structural abnormalities, and germline deletion did not cause early embryonic lethality. The discrepancy between our findings and those of Hagel and colleagues may stem from several factors. One key difference is the genetic background: Hagel et al. used 129/Sv or 129/Sv;C57BL/6 hybrid mice, while our study was conducted on a C57BL/6 background. Additionally, although the mutant alleles were engineered differently in the two studies, both result in a loss-of-function protein. Therefore, the contrasting phenotypes are likely attributable, at least in part, to differences in genetic background.

Here we found that loss of *Pxn* leads to heart, thymus and parathyroid defects. In particular, we focused on pharyngeal endoderm anomalies observed in *Pxn*^*−/−*^ embryos. Previous findings by other groups identified a role of FAs in the epithelial remodeling of pharyngeal pouch cell rearrangements [26, 27]. However, in our mutants, accumulation of endodermal cells in the PA suggests a defect in correct positioning of epithelial cells and formed multilayers of stratified epithelium but they still express E-cadherin and therefore they retain at least some epithelial properties. Loss of polarity in the absence of PXN has also been noted in epithelial mammary gland cells [3]. While further work will be necessary to establish in detail the cellular function of PXN in endodermal cells, our data suggest that *Pxn* is necessary for the morphogenesis of the organs derived from the third pharyngeal pouch. The deletion of *Pxn* in the *Tbx1* expression domain, which includes part of the third and fourth PA endoderm, mesoderm and ectoderm but not the NCCs, did not have phenotypic consequences. Thus, either the extent of recombination driven by *Tbx1*^*Cre*^ is insufficient, or the loss of *Pxn* also in the NCC is necessary to generate the observed phenotypic anomalies. Furthermore, a difference in the expression timing of *Tbx1* and *Pxn* in the pharyngeal tissues may account for the absence of a pharyngeal phenotype upon conditional deletion of *Pxn* in the *Tbx1*-expressing domain. Literature information about the exact timing and regionalization of *Pxn* expression relative to *Tbx1* expression is limited. Hagel et al., [4] showed that at E8.5 paxillin expression was detected in the mesodermally and endodermally derived structures. However, single-cell RNA-seq datasets from developing E8.5 mouse embryo (https://marionilab.cruk.cam.ac.uk/MouseGastrulation2018/) reveals that *Pxn* is broadly expressed across multiple tissue types, with relatively low expression levels in the endoderm; conversely *Tbx1*expression appears to be restricted primarily to the mesoderm, with limited expression in the endoderm.

*Tbx1* heterozygosity in a *Pxn* null background caused cardiac septation and 3PP-derived abnormalities. In contrast, *Tbx1Cre*, *Mef2c-AHF-Cre-* and *Mesp1Cre*-driven deletion did not lead to any structural defects. Thus, *Pxn* appears to be dispensable in SHF cells. These findings do not exclude a role of *Pxn* in the SHF because loss of *Pxn* might be compensated by *Hic5,* a *Pxn* paralog gene. In fact, *Hic5* expression in the SHF but not in endoderm and in NCC, both tissues contributing to pharynx development, is consistent with the absence of cardiac defects in *Pxn* conditional deleted embryos.

Our data show that heterozygous deletion of *Tbx1* enhances significantly the penetrance of developmental defects in *Pxn* heterozygous mice, suggesting an interaction between the two genes,

Published data from cellular and mouse models indicate that TBX1 is a transcriptional regulator of *Pxn* gene expression [8, 11], providing a possible rational to explain the interaction.

Pharyngeal endoderm-specific deletion of *Tbx1* causes absence of the thymus and parathyroids [28, 29], similarly to *Tbx1*^*−/−*^ embryos, indicating that *Tbx1* in the pharyngeal endoderm is required for patterning and development of the pharyngeal pouches. Furthermore, *Tbx1* itself might regulate the morphogenesis of the epithelial layer of the PA [27, 30, 31]. *Ripply3* gene, a direct target of *Tbx1*, is required for the epithelial architecture of the pharyngeal pouches during the development of the PA [27, 31]. Accordingly, it has been shown a mechanism regulating the epithelial identity by Ripply3 protein accumulating in the FA, which would allow it to transmit mechanical force during the bending of the pharyngeal epithelial layer [27, 31].

Here we propose a mechanism of pharyngeal pouch morphogenesis in which *Pxn* has a structural role in the pouch morphogenesis. The decreased size of 3PP of *Pxn* mutants led us to hypothesize that the absence of PXN has an effect on mechanical force upon the epithelium for the morphogenesis of the pharyngeal endoderm, given that it is known that the epithelial endodermal bending implies a mechanical stress [26, 27]. We speculate that the pharyngeal endoderm defects may result from anomalies in integrin turnover and/or altered rates of focal adhesion disassembly cause by the absence of *Pxn* [10].

In summary, we found that loss of *Pxn* expression impacts the organization of the 3PP endoderm, where we observed a loss of *Gcm2* expression. Consistently, we observed severe developmental anomalies of the endodermally derived thymus and parathyroids.

Interestingly, 22q11.2DS patients, who are heterozygously deleted for *TBX1 *[32], have anomalies within structures derived from the PA and are often affected by hypocalcaemia and hypoplasia of parathyroids, and therefore it would be of interest to determine whether variants of the *Pxn* gene affect the penetrance of this clinically relevant phenotype.

## Materials and methods

### Mouse lines

The *Pxn*^*fl/fl*^ mice were generated as previously described [3, 14] and were maintained in a clean facility in a C57Bl6 background. *Tbx1*^*lacZ/*+^ (also indicated here as *Tbx1*^*−/*+^), *Tbx1*^*Cre/*+^, *Mesp1*^*Cre/*+^, *Mef2c-AHF-Cre* are available through the EMMA/Infrafrontiers repository under the codes: EM:02137, EM:11399, EM:15684 respectively. Genotyping was carried out according to instructions provided by the original report. Embryos were collected at E8.5, E9.5 or E18.5; for timed crosses, developmental stage was evaluated by considering the morning of vaginal plug as embryonic (E) day 0.5, and by counting somites of embryos. Animal studies were carried out according to the animal protocol 257/2015-PR (licensed to the AB lab) reviewed by the Italian Istituto Superiore di Sanità and approved by the Italian Ministero della Salute, according to Italian regulations.

### Histology and immunofluorescence

Hearts were dissected, isolated from near-term pups (E17.5, E18.5) and then embedded in paraffin, sectioned and stained with Hematoxylin–Eosin according to standard protocols. E9.5 (23–25 somites) mouse embryos were fixed in 4% PFA and embedded in OCT. 10 μm sagittal or transverse cryosections were subjected to immunofluorescence (IF) using primary antibodies (in Table 2, incubation overnight at 4 °C) and incubated with the appropriate secondary antibody labelled with a fluorescent probe (for 1 h. Dilution 1:400). Alternatively, E10.5 (30–34 somites) mouse embryos were embedded in paraffin. For IF analysis 10 µm coronal sections were deparaffinized in xylene, rehydrated, and, after antigen unmasking with citrate buffer; then, sections were incubated overnight at room temperature with primary antibodies (in 0.5% milk, 10% fetal bovine serum, 1% bovine serum albumin in H_2_O) (in Table 2) and the secondary antibodies were incubated with sections for 1 h at room temperature. The nuclei were counterstained with DAPI. Confocal images were acquired with an inverted confocal microscope (NikonA1).

**Table 2:** Primary antibodies used for immunofluorescence analysis

Pxn	Rabbit	Abcam #ab32084
E-cadherin	Mouse	BD #610182
Integrin β1 total	Rat	Millipore MAB1997
CD29	Rat	BD #553715
Pospho-Histone H3 (Ser10)	Rabbit	Millipore #06–570
Ap2alpha	Mouse	DSHB #3B5-c
Hic5	Mouse	BD #611164
Tbx1	Rabbit	Abcam #ab18530

### RNAscope

*RNAscope on tissue sections:* After incubation of 4% PFA at RT overnight, embryos were processed with 25%, 50%, 75%, 100% Methanol for 30 min at RT. Embryos were embedded in paraffin and sectioned at 5 ± 1 µm thickness. The sections were processed with RNAscope® Multiplex Fluorescent v2 reagents (Advanced Cell Diagnostics, Cat# 323100), according to the manufacturer’s instructions. Sections were heated at 60 °C for an hour, then were deparaffinized in xylene and dehydrated in 100% ethanol. Sections were incubated with RNAscope™ hydrogen peroxide (Cat No. 322335) at RT in 10’. The sections were incubated in boiled 1 × Target retrieval reagent, for 15’. After rinsing with distilled water and 100% ethanol, the sections were dried at RT. The sections were incubated with Protease Plus (RNAscope™ Protease III Cat No. 322337) at 40 °C for 15’. After rinsing with distilled water, the sections were incubated for two hours at 40 °C with RNA probe of C1 channel (RNAscope™ Probe-Mm-Tbx1 Cat No. 481911). After hybridization, the amplification with TSA was performed: AMP1 at 40 °C for 30 min, add AMP2 at 40 °C for 30 min and AMP3 at 40 °C for 15 min. The sections were then washed twice with Wash Buffer1x (RNAscope™ Wash Buffer Cat No. 310091) for two minutes at RT; HRP-C1 was added for 15 min at 40 °C for marking C1 probe. Then, TSA-FITC was incubated for 30 min at 40 °C and then HRP-blocker for 15 min at 40 °C.

Immunofluorescence: at the end of the procedure the sections were processed by immunofluorescence. The sections were rinsed with TBS-10% tween-20. Following the blocking in TBS-0.1% BSA + 10% Sheep serum for 1 h at RT, the sections were incubated in TBS-0.1% BSA with Hic5 (BD Cat: 611164) overnight at RT. Sections were than incubated with the secondary antibody for 1 h at RT in TBS-0.1% BSA. Then, sections were incubated with TSA-CY3 1:300 for 15 min at RT. The slides were mounted with ProLong anti-Fade with DAPI.

Images were acquired using an inverted confocal microscope (NikonA1).

### In situ hybridization

For in situ hybridization, antisense RNA probes used were used for Gcm2 and FoxN1. The probes were labelled using a digoxigenin RNA and fluorescein RNA labelling kit, respectively. E11.5 mouse embryos were cryoprotected by serial dilution of sucrose/1 × PBS (10%, 20% and 30% sucrose) at 4 °C, then incubated for 2 h at 4 °C in 50:50 v/v 30% sucrose//OCT prior to embedding in OCT, then embedded in OCT compound and sectioned into 10 µm transverse or sagittal sections. To perform the in situ hybridization at E16.5, 10 µm coronal cryosections were incubated with the probes overnight at 70 °C, and then incubated with primary antibody overnight at 4 °C. Slides were then incubated with a chromogenic substrate for alkaline phosphatase, until the signal had developed. The images were acquired using a Nikon Automatic Microscope with 10 × and 20 × objectives. The area of the expression domains of the individual markers was calculated using *ImageJ* software.

### Quantitative analysis of the pharyngeal endoderm

We analyzed the cells surrounding the pharyngeal arches, positive for anti-E-cadherin antibody (which marks the epithelial cells). In order to quantify the number of positive cells, confocal images were processed through the *ImageJ* software and E-cadherin/DAPI double positive cells were counted. Subsequently, the average of number of cells counted for each pharyngeal arch was plotted.

### Quantitative analysis of Tbx1 level in RNAscope experiments

For *Tbx1*, optical density (OD) was measured from 2D images using the ImageJ graphic pen tool. Background-corrected OD values were obtained by subtracting the slice background OD from the measured signal within defined regions of interest (ROIs) of 50 μm^2^. Measurements were taken around the pharyngeal arches (PAs) and the second heart field (SHF). Quantification was based on RNAscope staining from five embryos (*N* = 5).

### Statistical analysis

Data are presented as mean ± SD. *n* represents number of animals. Differences were considered statistically significant when *p* < 0.05 (Graph Pad Software).

To evaluate the statistical significance of cardiac and pharyngeal phenotypes (Table 1) we used Fisher's exact test. To test statistical significance of 3PP for all genotypes analysed we used two-tailed Student's t-test. To evaluate cell proliferation in *Pxn*^*−/−*^ vs *WT* we counted E-cadherin + and E-cadherin +;P-H3 + cells in the pharyngeal arches of the embryos. All experiments were performed at least on 3 embryos per genotype. Data were subjected to normalizing transformation and evaluated using two-tailed Student's t-test.

## Supplementary Information

Below is the link to the electronic supplementary material.

Supplementary file1 (PDF 14.4 MB)

## Data Availability

All data produced or examined during this investigation are comprehensively presented in this published article, its supplementary information files.
