# Supplementary material for: Paxillin is crucial for thymus and parathyroid development by regulating the architecture of the third pharyngeal pouch endoderm
Source: Cell Mol Life Sci. 2026 Jan 26;83(1):68. doi: 10.1007/s00018-025-05973-6 (PMC12847588; doi:10.1007/s00018-025-05973-6)

Supplementary Fig.1

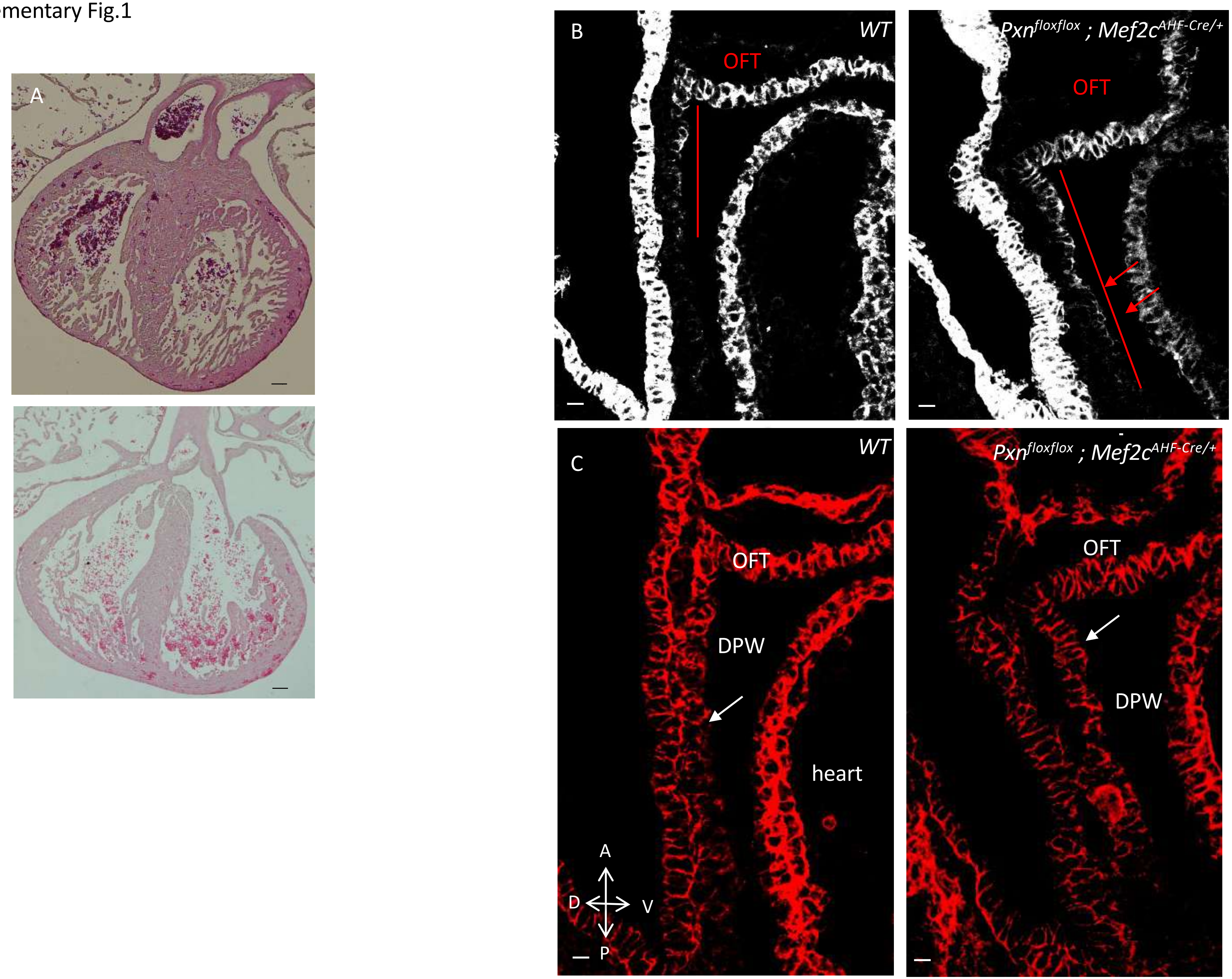

Supplementary Fig.2

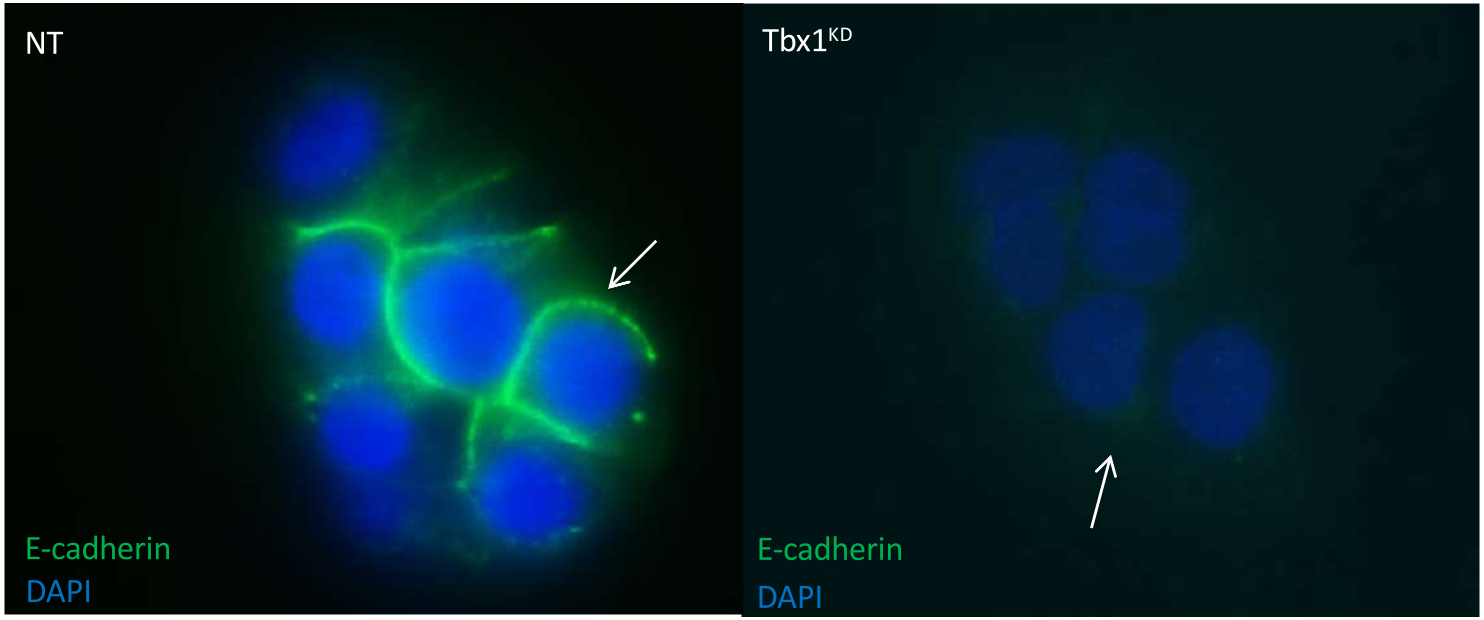

Supplementary Fig.3

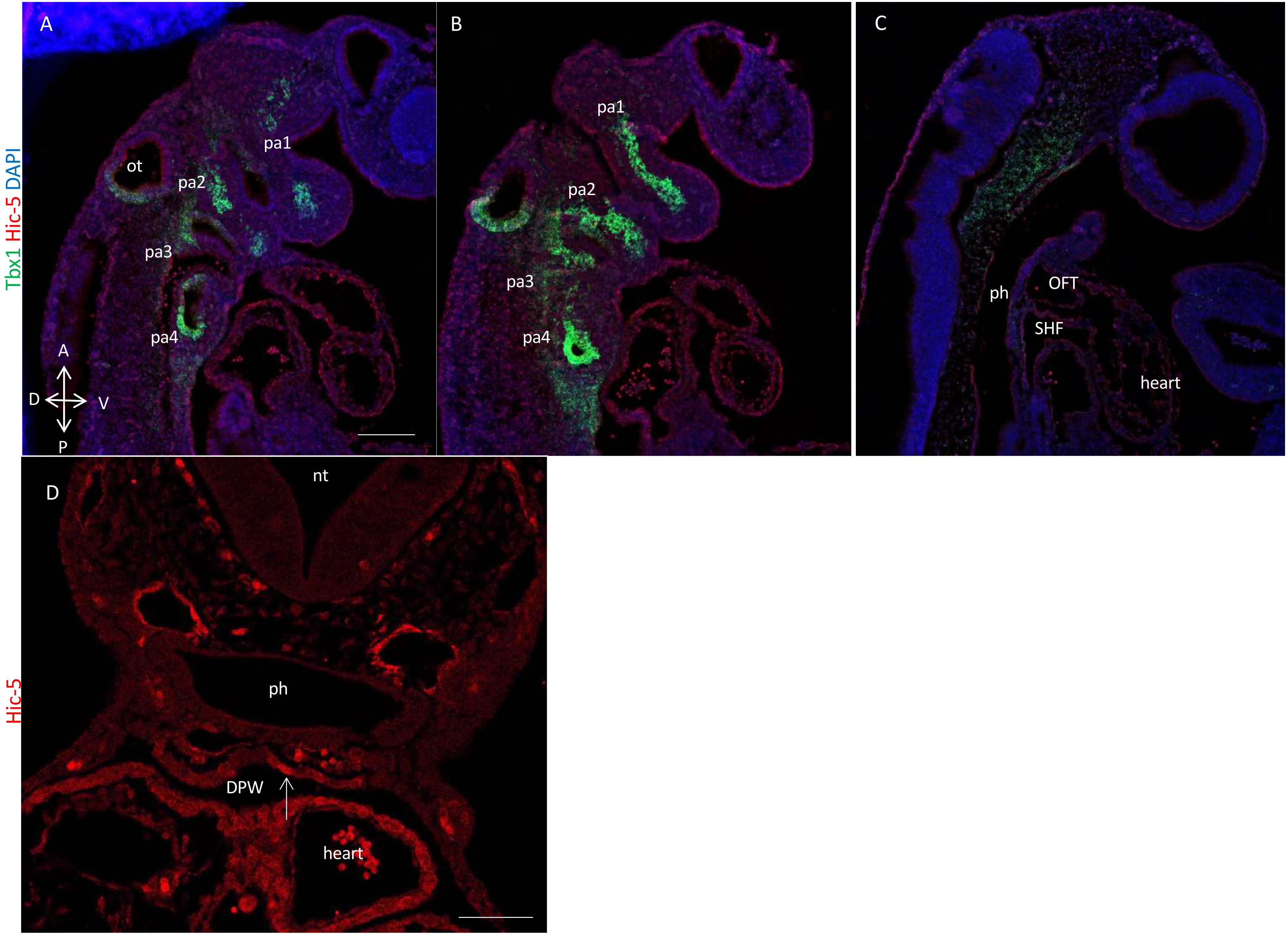

Supplementary Fig.4

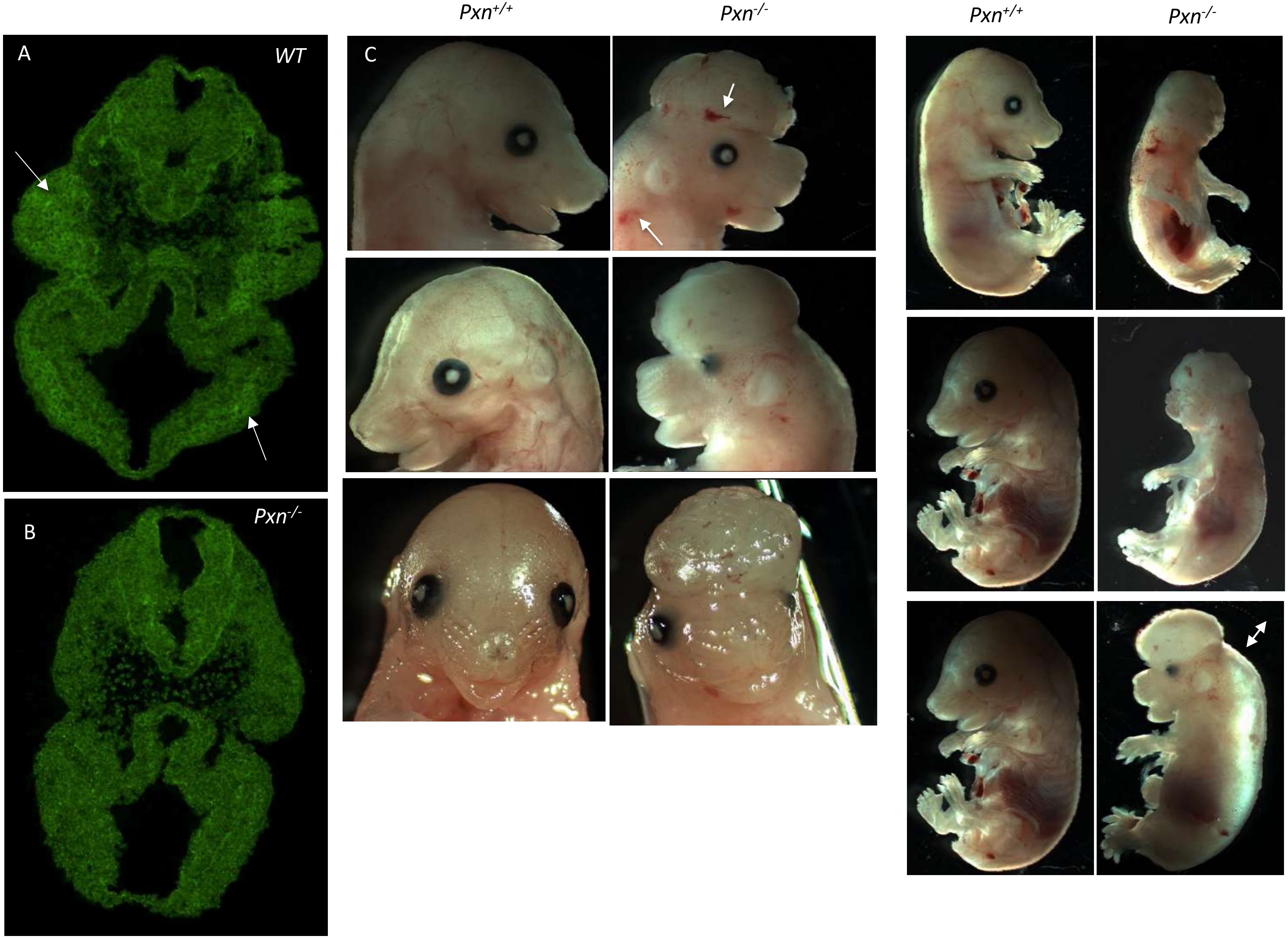

Supplementary Fig.5

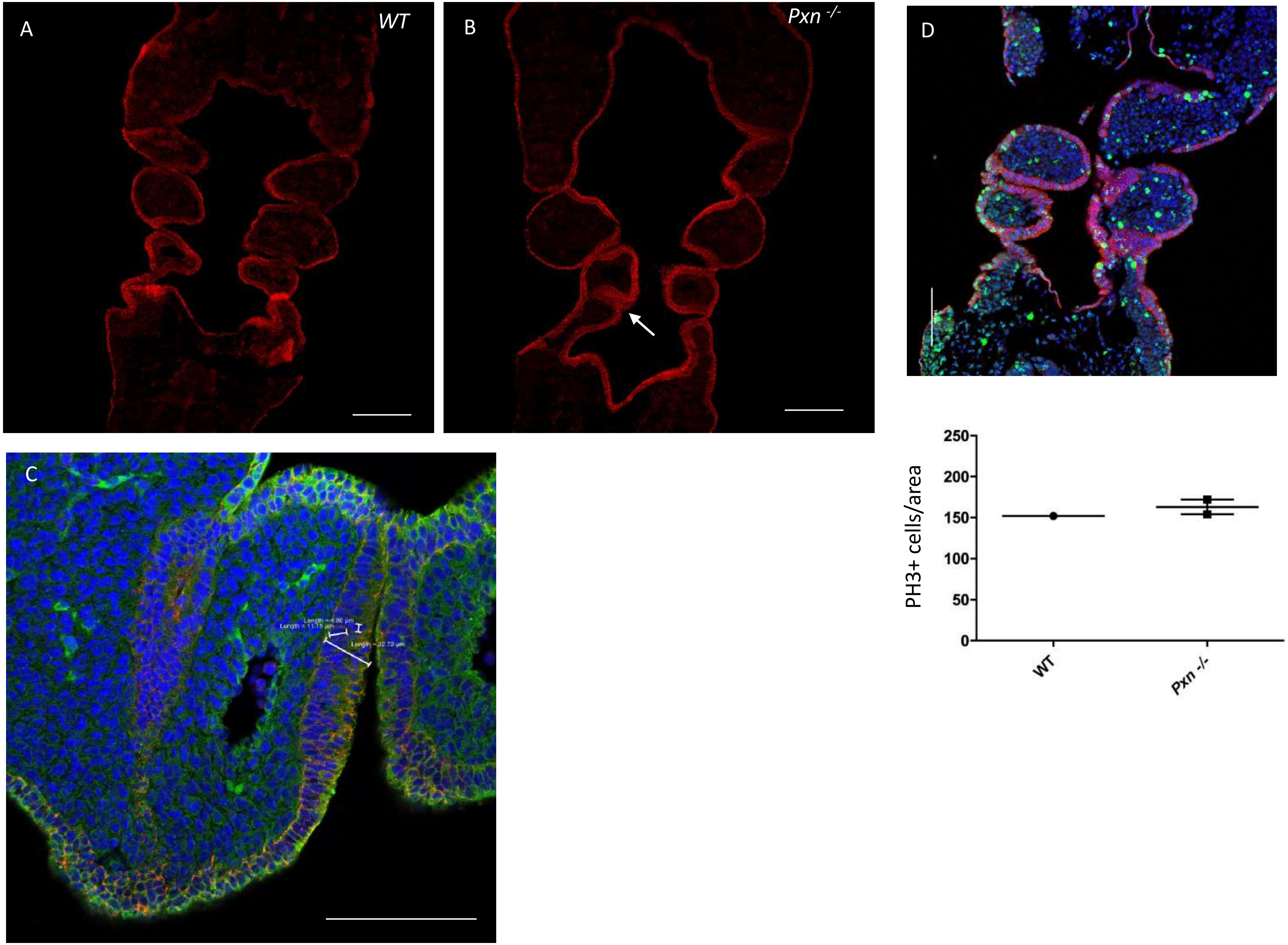

Supplementary Fig.6

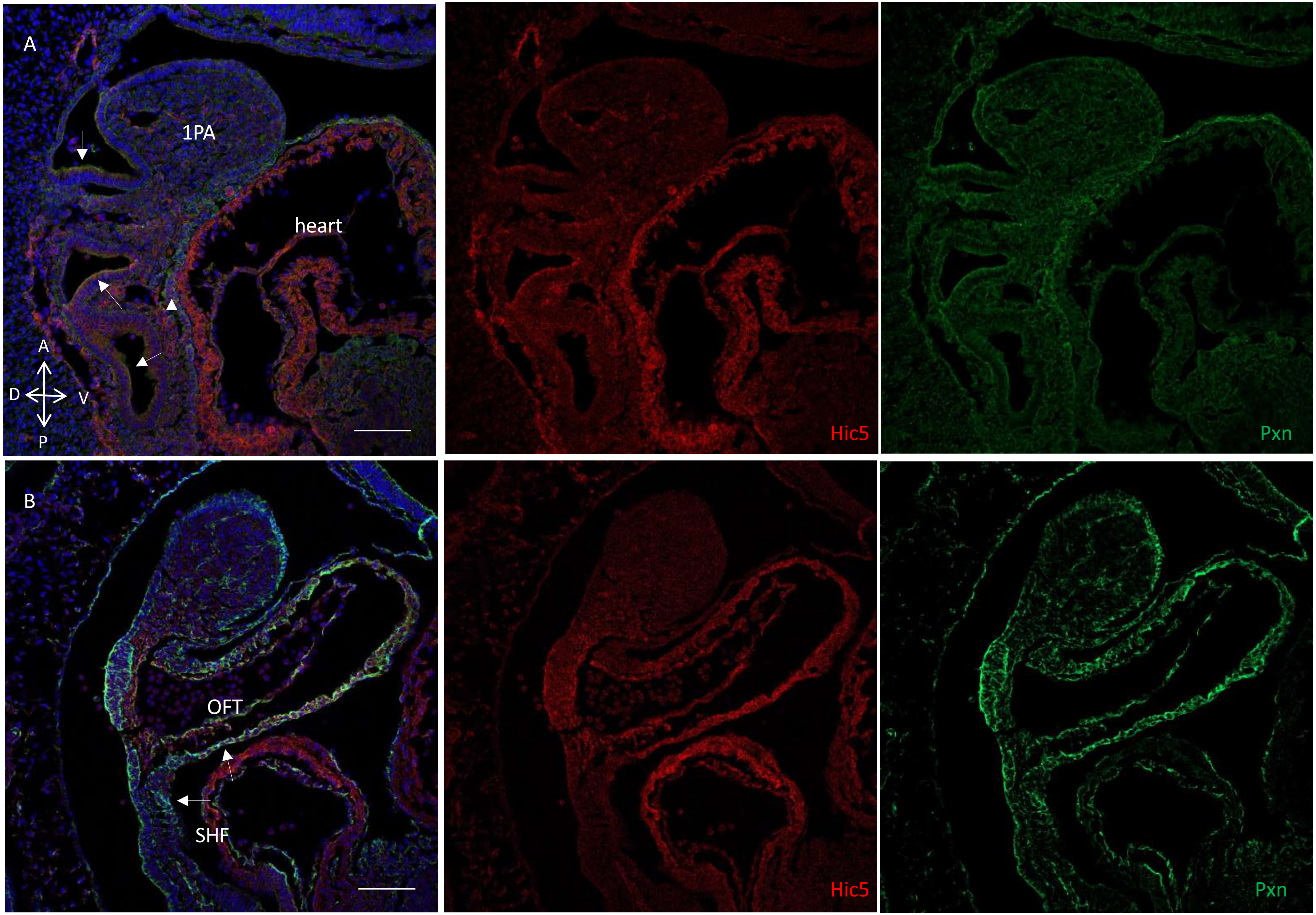

Supplementary Fig. 7

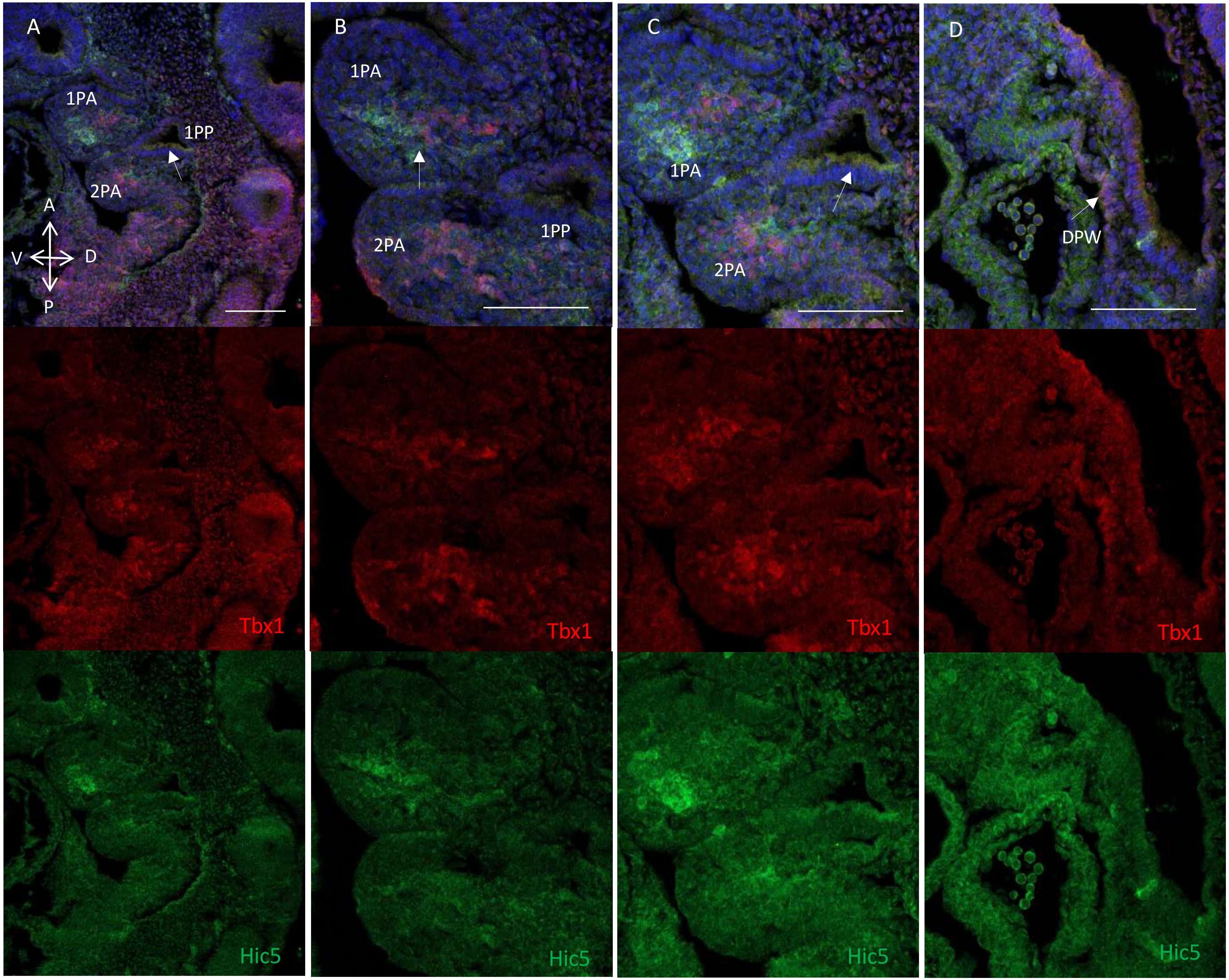

Supplementary Fig.8

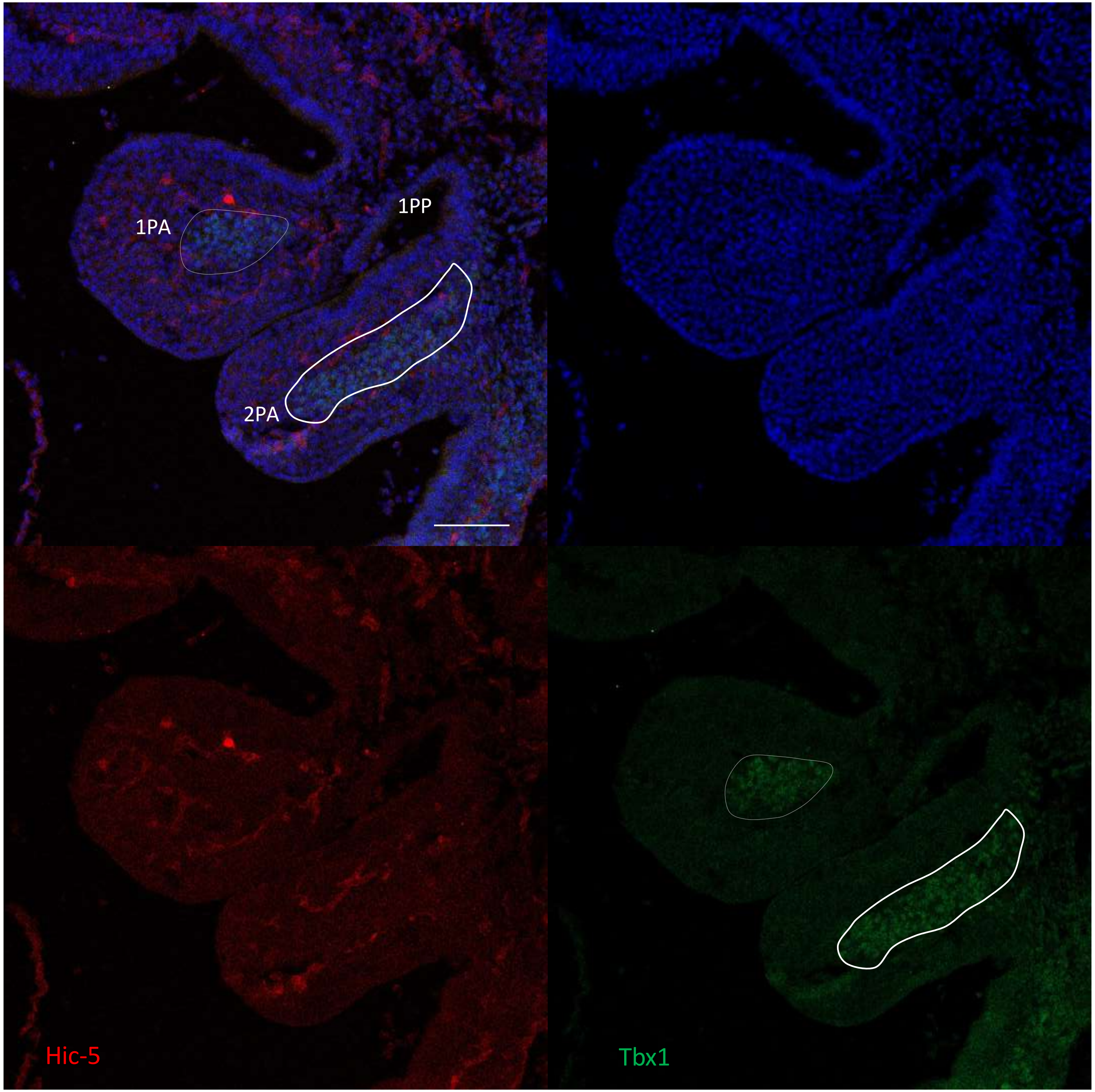

Supplementary Fig.9

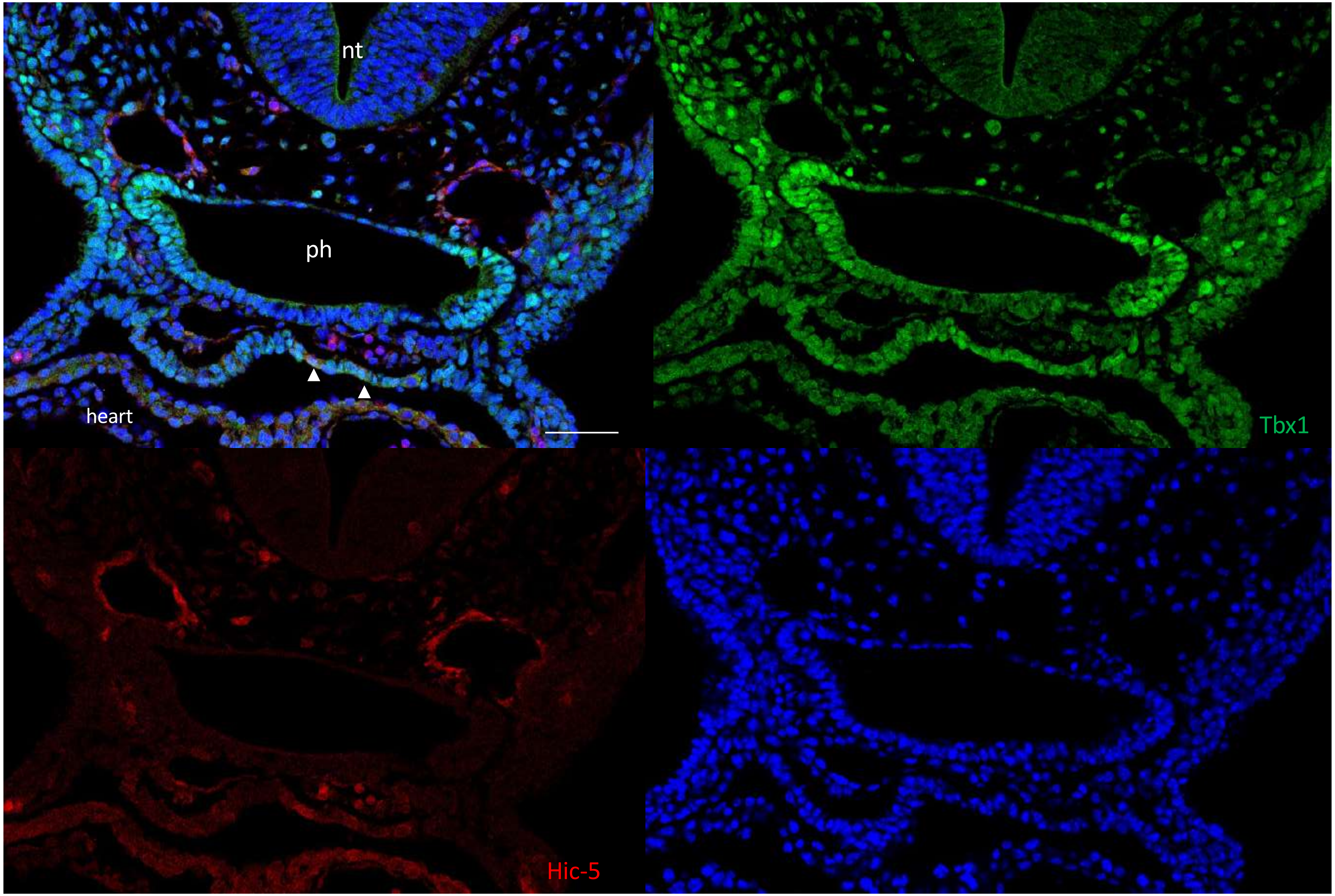

Supplementary Fig.10

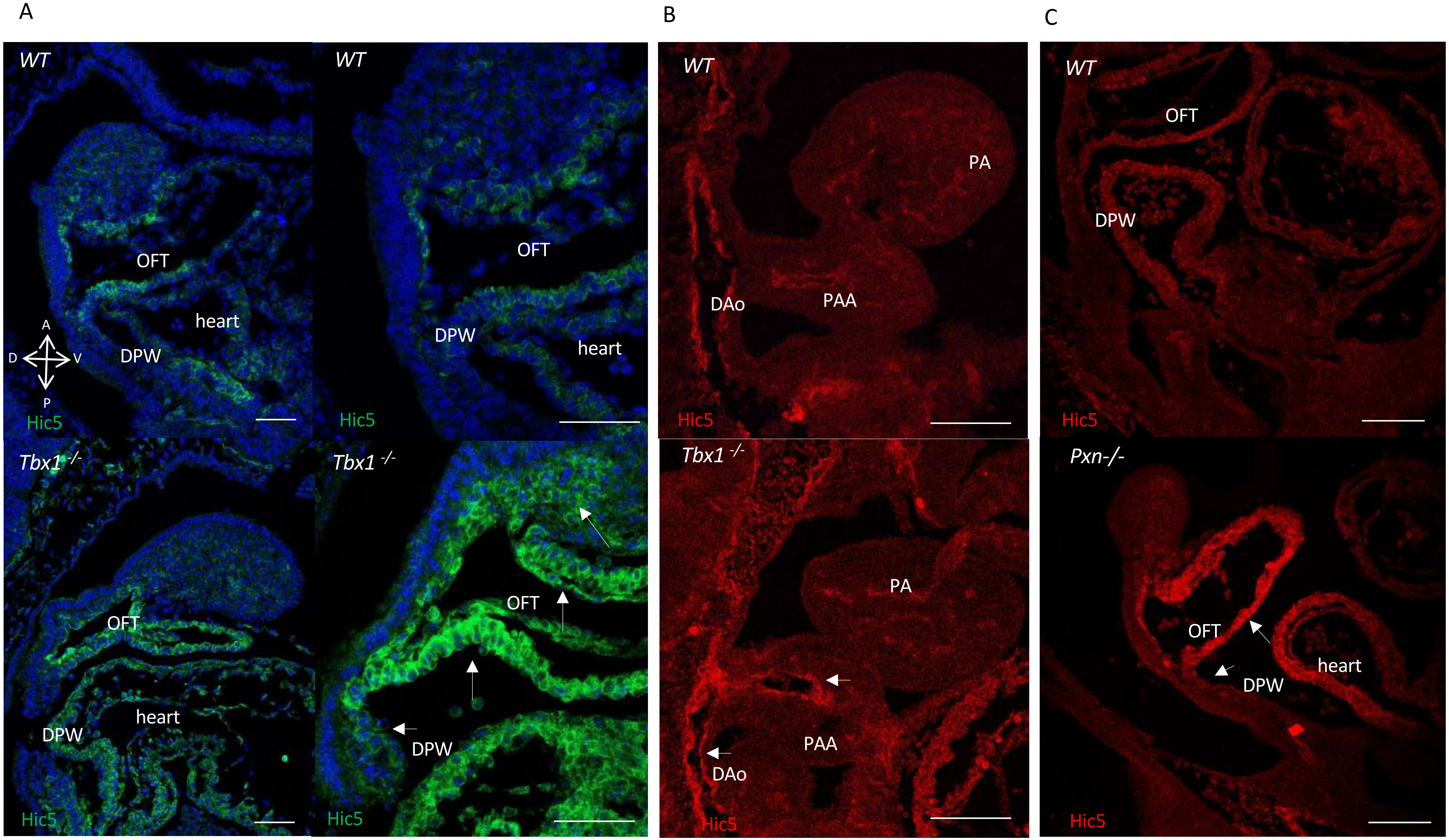

Supplementary Fig.11

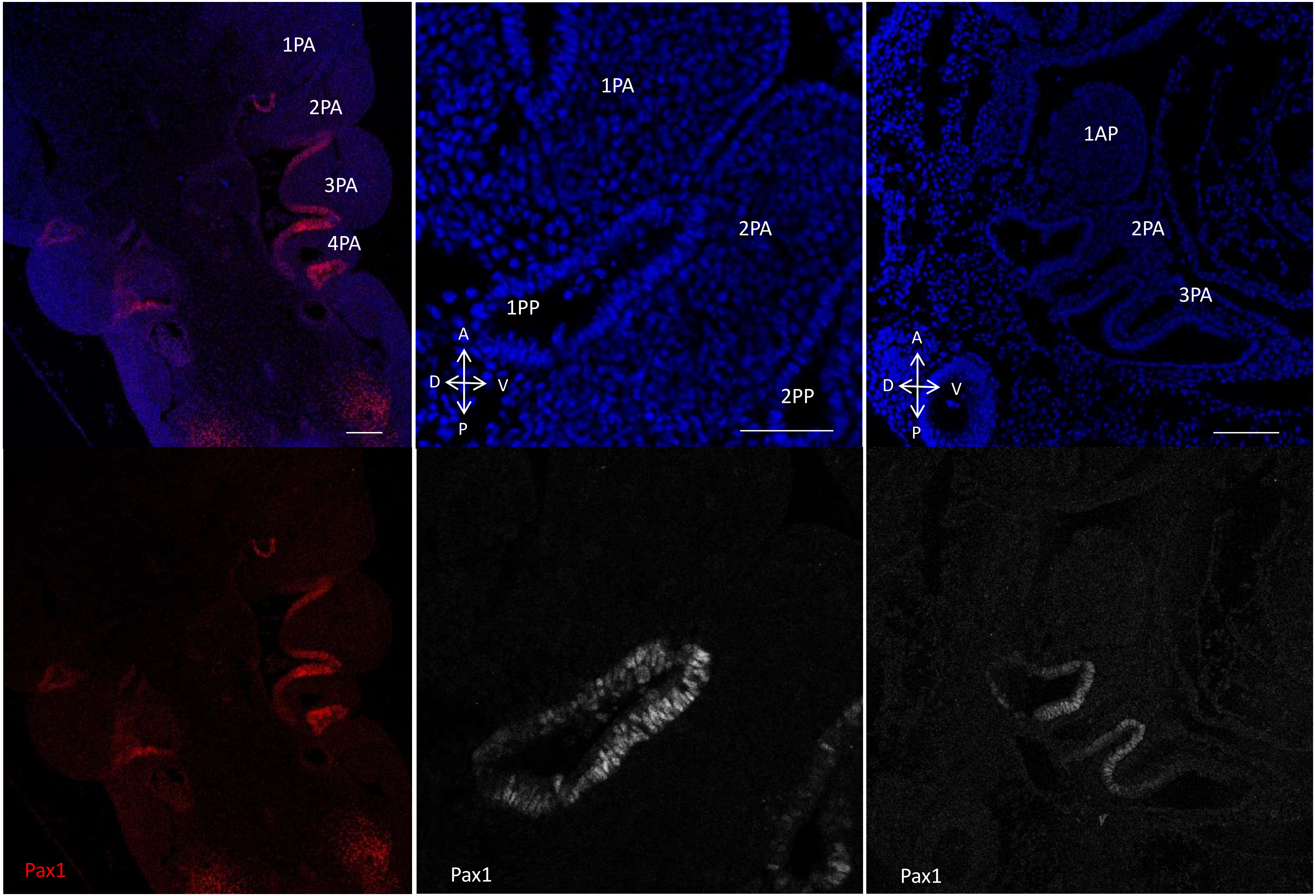

Supplementary Fig.12

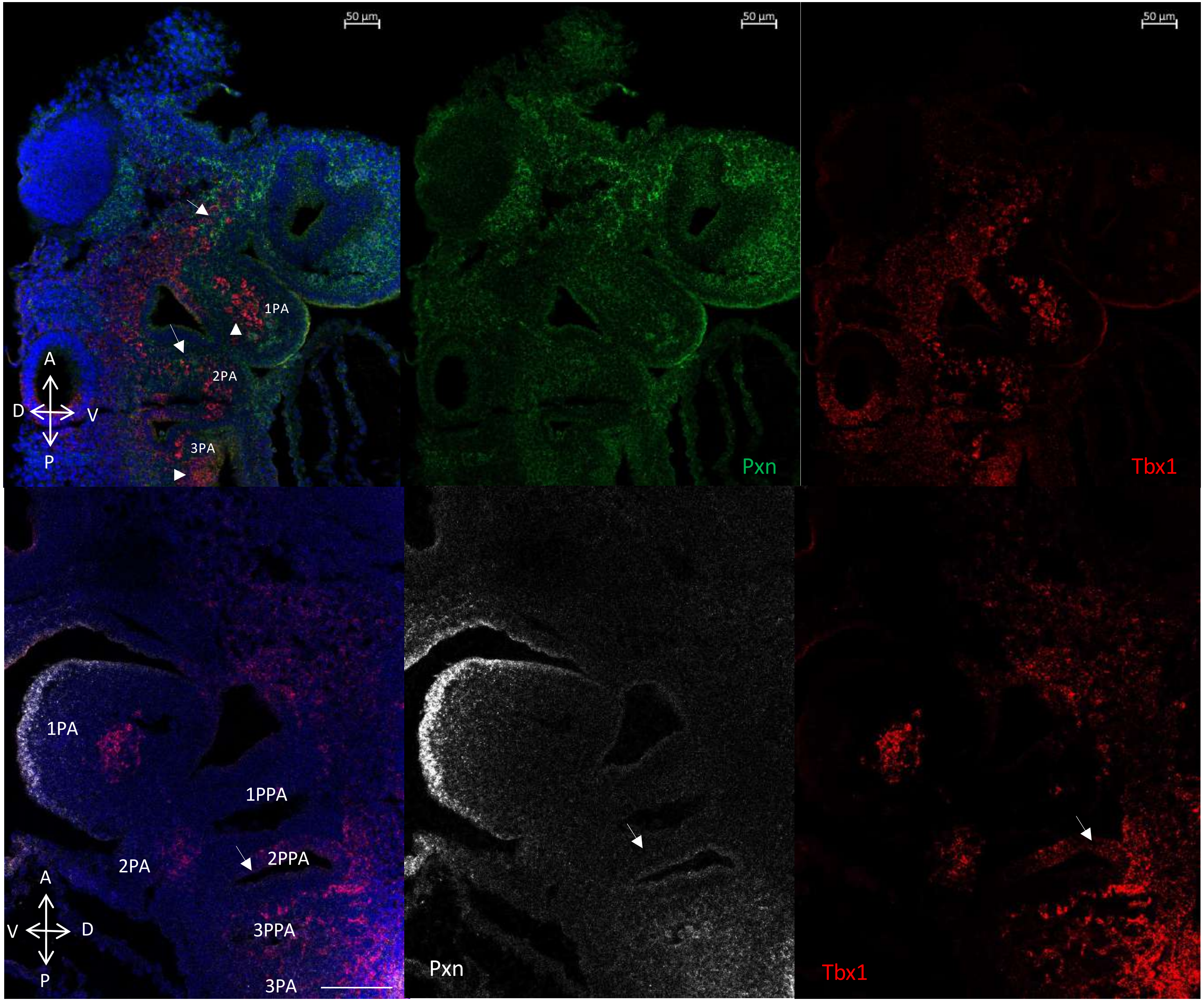

Supplementary Fig.13

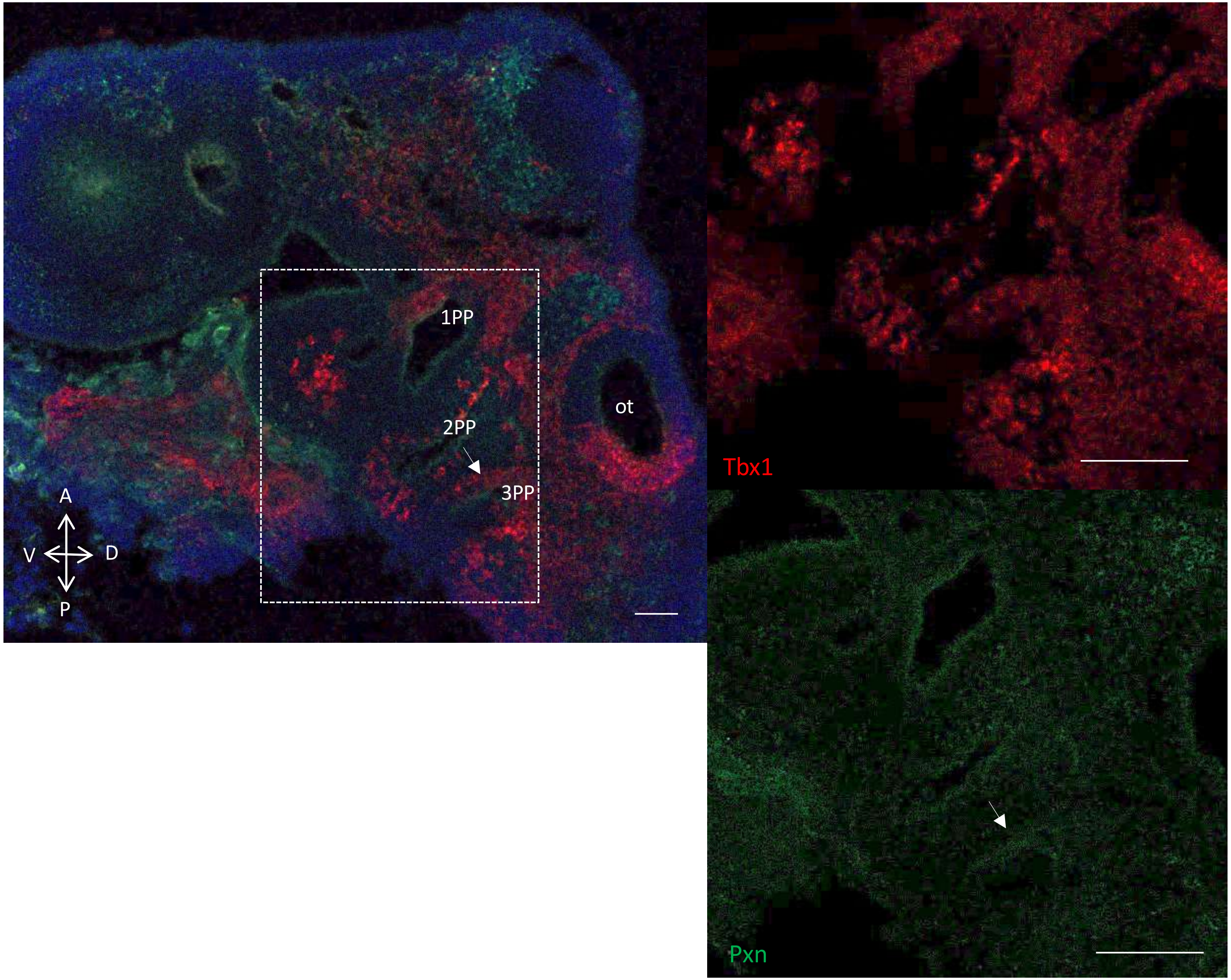

Supplementary Fig.14

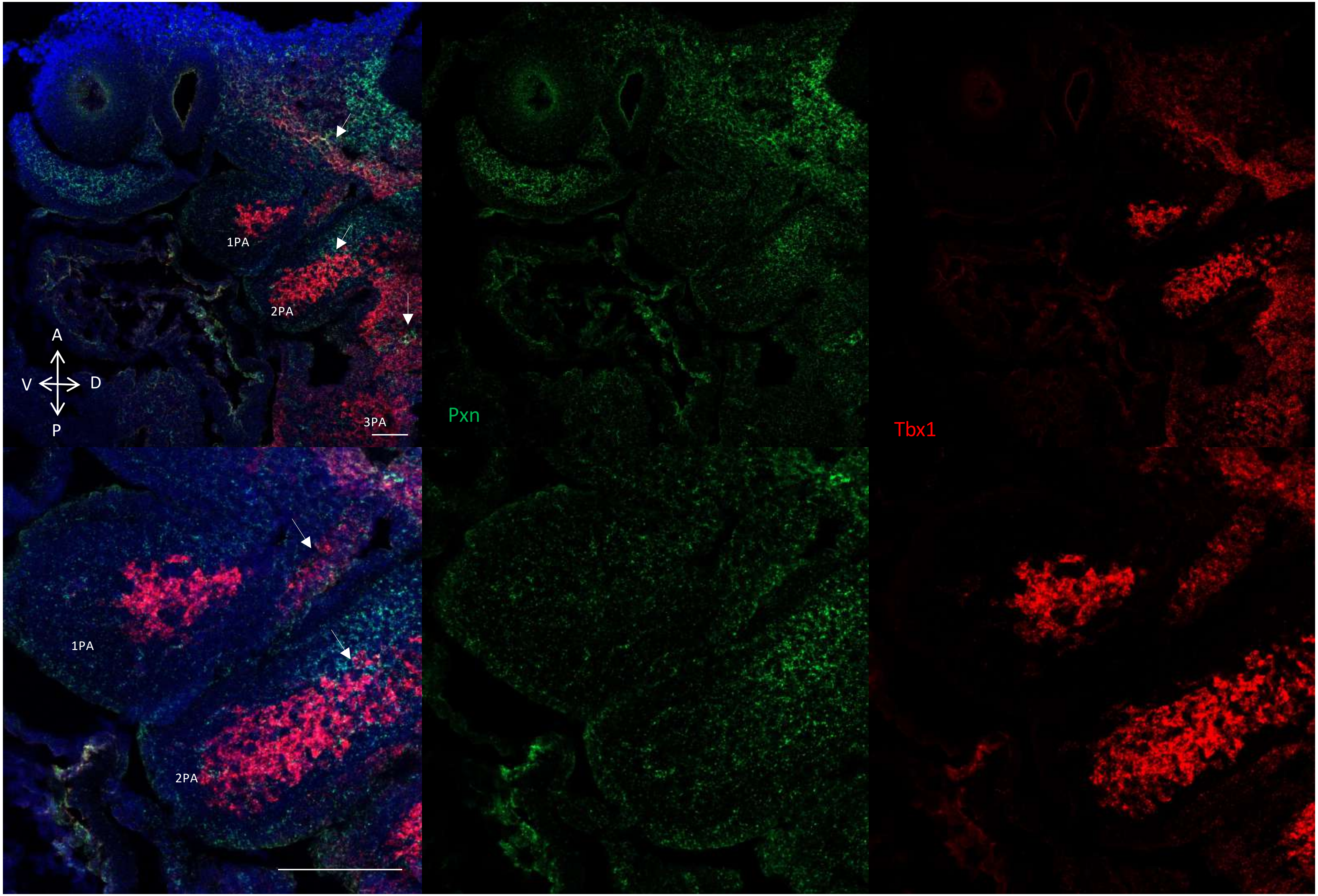

Supplementary Fig.15

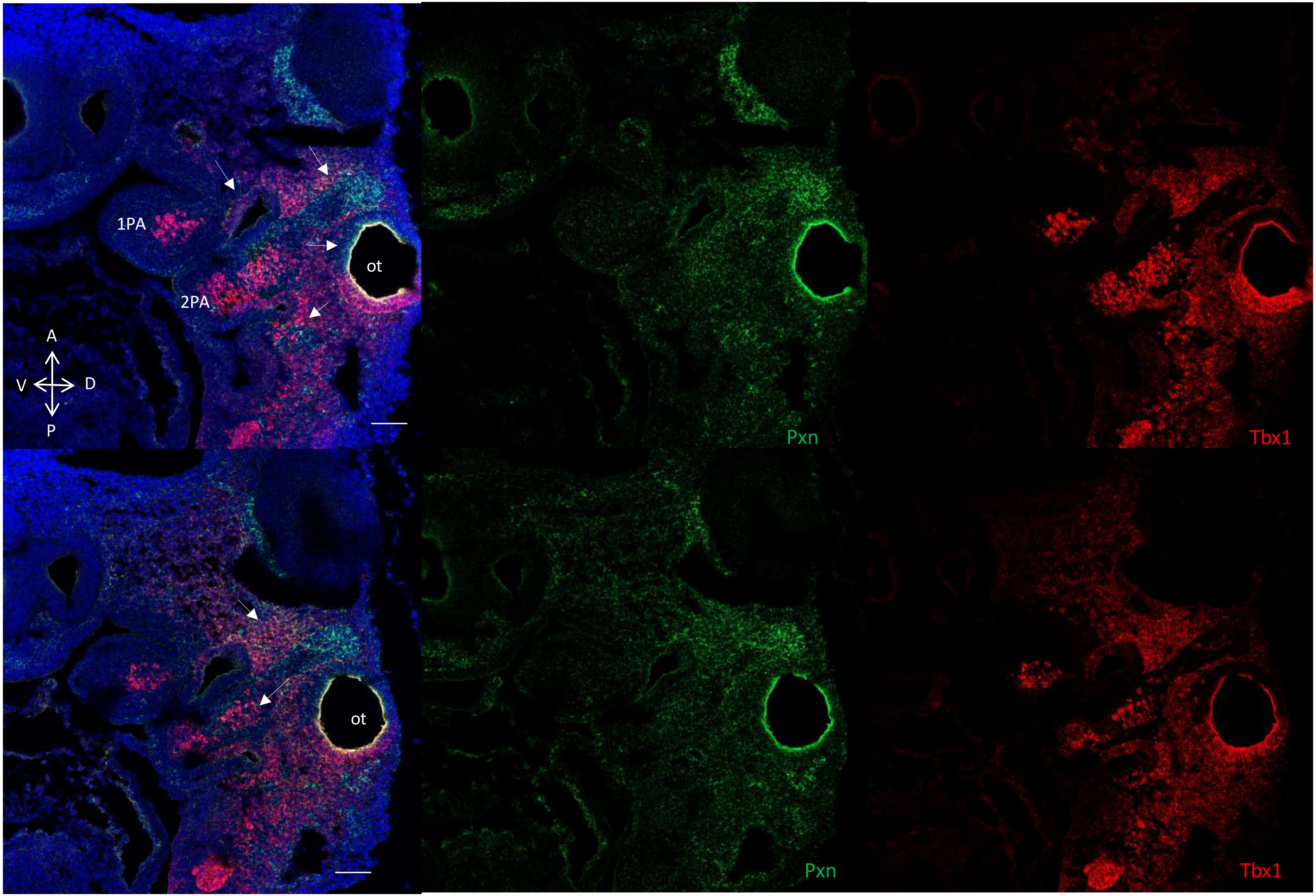

Supplementary Fig.16

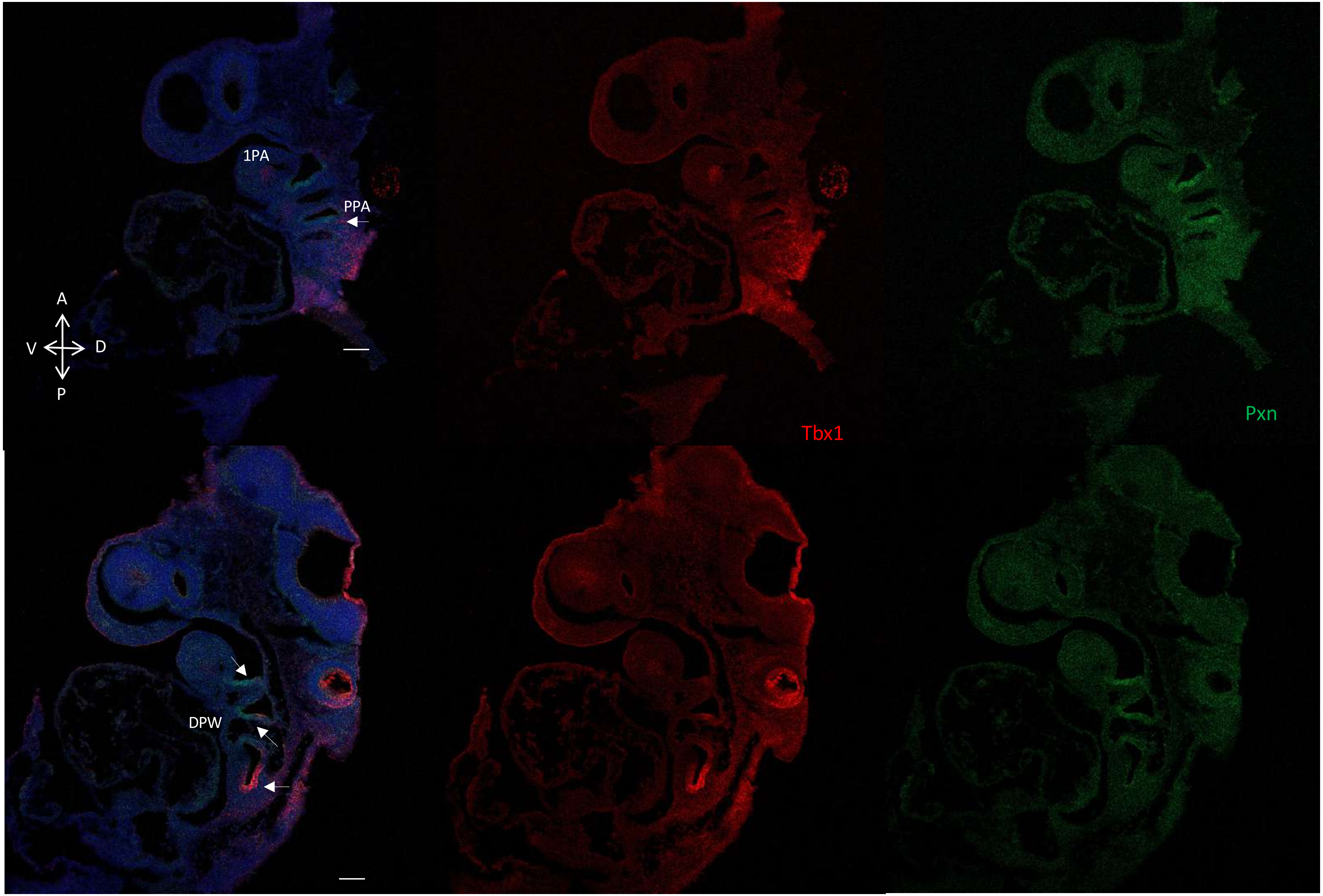

Supplementary Fig.17

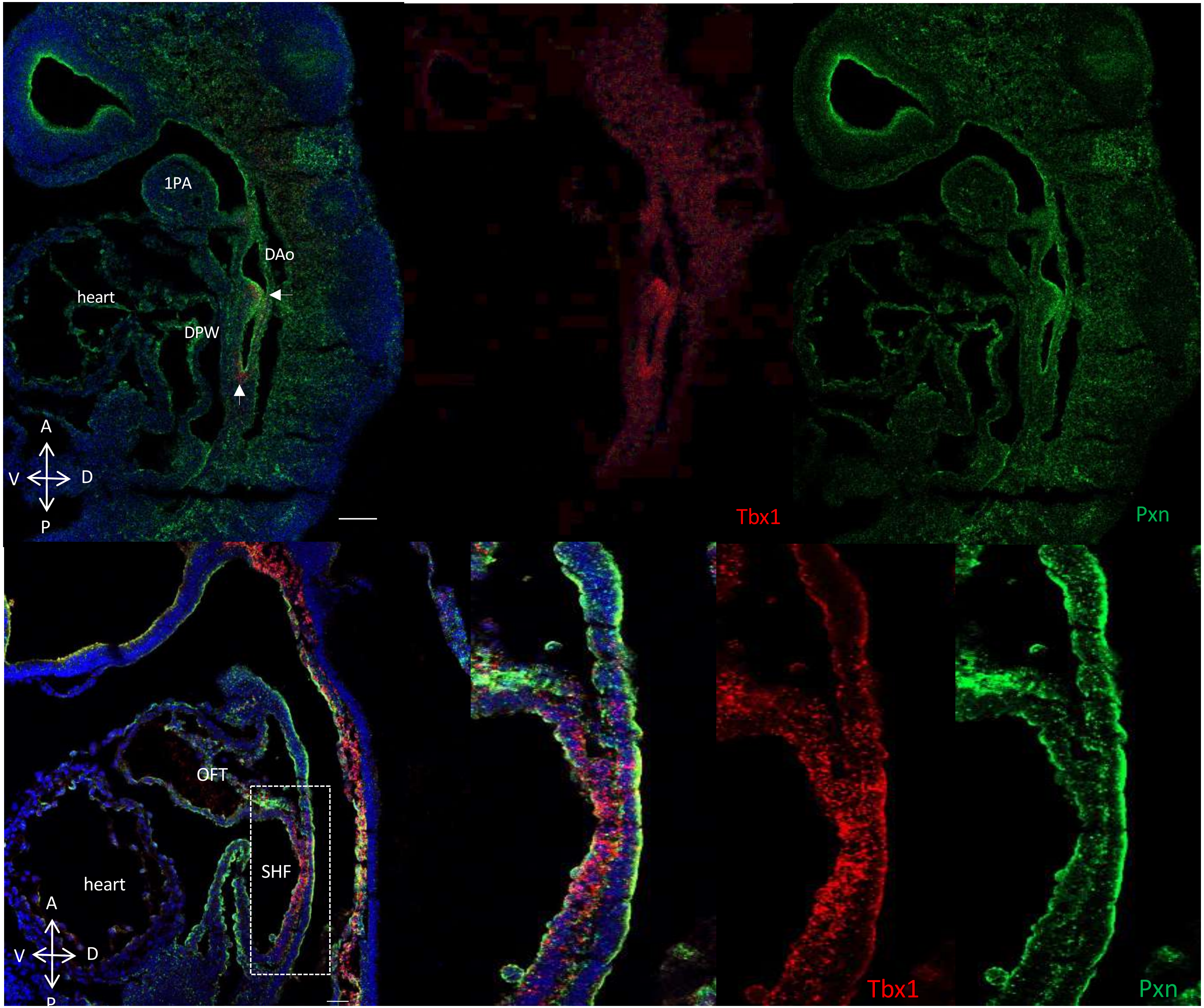

Supplementary Fig.18

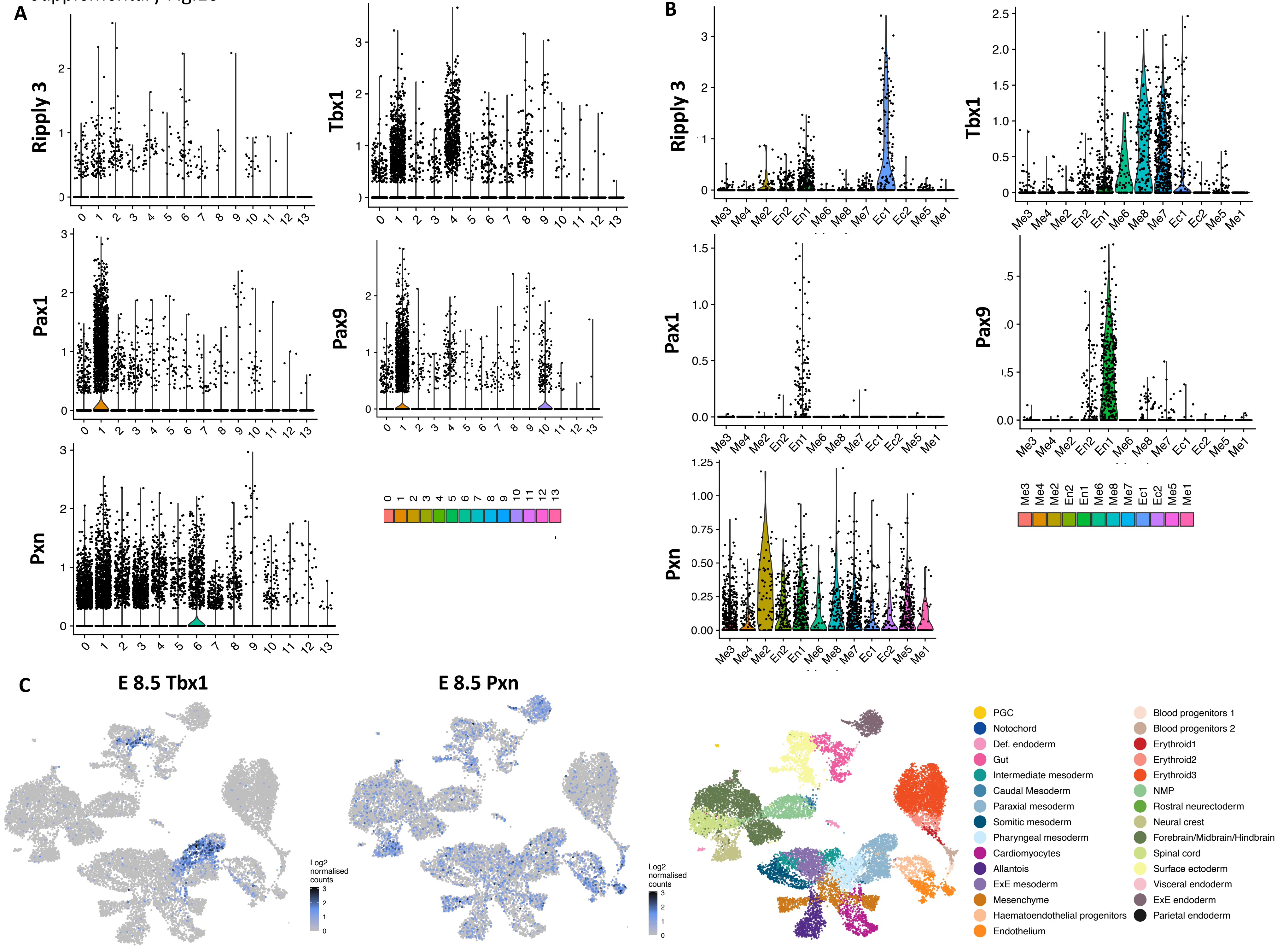

Supplementary Fig.19

A

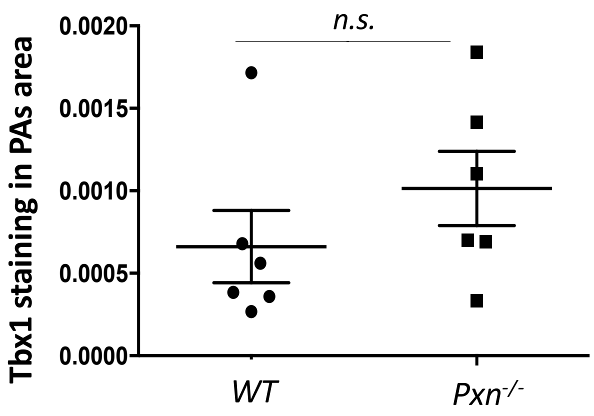

B

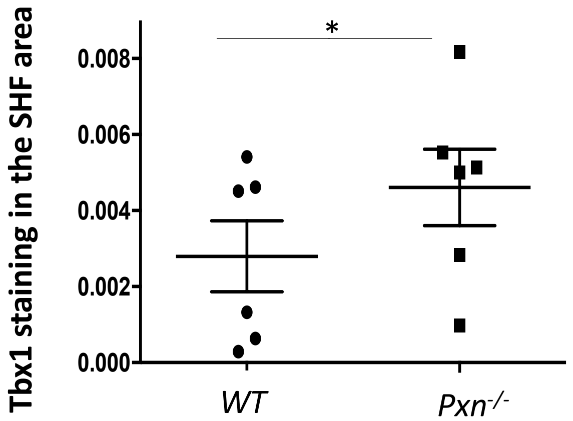

Supplement: Supplementary file 1 — Supplementary file1 (PDF 14.4 MB) [file 18_2025_5973_MOESM1_ESM.pdf]
